# Supplementary material for: Cross-sector user and provider perceptions on experiences of shared-care clozapine: a qualitative study
Source: BMJ Open. 2017 Sep 28;7(9):e017183. doi: 10.1136/bmjopen-2017-017183 (PMC5640131; doi:10.1136/bmjopen-2017-017183)
Supplement: Author's manuscript [file bmjopen-2017-017183.draft_revisions.pdf]

BMJ Open is committed to open peer review. As part of this commitment we make the peer review history of every article we publish publicly available.

When an article is published we post the peer reviewers' comments and the authors' responses online. We also post the versions of the paper that were used during peer review. These are the versions that the peer review comments apply to.

The versions of the paper that follow are the versions that were submitted during the peer review process. They are not the versions of record or the final published versions. They should not be cited or distributed as the published version of this manuscript.

BMJ Open is an open access journal and the full, final, typeset and author-corrected version of record of the manuscript is available on our site with no access controls, subscription charges or pay-per-view fees (http://bmjopen.bmj.com).

If you have any questions on BMJ Open's open peer review process please email info.bmjopen@bmj.com

Enabling person-centred care through shared-care clozapine

Journal:	*BMJ Open*
Manuscript ID	bmjopen-2017-017183
Article Type:	Research
Date Submitted by the Author:	12-Apr-2017
Complete List of Authors:	Sowerby, Camilla; Universtiy of Bath, Pharmacy and Pharmacology<br>Taylor, Denise; Universtiy of Bath, Pharmacy and Pharmacology
**Primary Subject Heading**:	Mental health
Secondary Subject Heading:	Qualitative research, Patient-centred medicine, Health services research, General practice / Family practice
Keywords:	MENTAL HEALTH, QUALITATIVE RESEARCH, Schizophrenia & psychotic disorders < PSYCHIATRY

SCHOLARONE™
Manuscripts

Title: Enabling person-centred care through shared-care clozapine

Mrs Camilla Sowerby and Dr Denise Taylor

Corresponding author: Mrs Camilla Sowerby, The Old School House School Lane Aston Rowant, OX49 5SU, Camilla.sowerby@berkshire.nhs.uk, 07876216828

Co-author: Dr Denise Taylor, University of Bath, Bath, UK

Key words: Shared-care, Clozapine service, Primary care, Cross sector working, Multi-professional working

Word count – 4939 words

Abstract

Objectives: To understand the effectiveness and acceptability of a shared-care clozapine service.

Design: Semi-structured interview and focus group methodology were used and analysed with interpretative phenomenological analysis. Ethical approval 13/EM/0286 was gained in July 2013.

Participants: Six different groups of healthcare professionals (HCPs) involved in shared-care clozapine provision were identified. and To be eligible for participation, HCPs needed to be involved with at least one shared-care clozapine service user (CSU). CSUs were eligible to participate as long as they currently received their clozapine through shared-care andcarers of shared-care CSUs were eligible to participate if the CSU they cared for agreed to invite them to participate in the research.

Results: 32 HCPs and 6 CSUs were recruited and 14 interviews and 6 participant homogenous focus groups were run. Four shared superordinate themes were identified; Clozapine Process, The Sharing of Care, The Provision of Care and Multi-professional Relationships. The Wish conceptual model of relationships provided further insight into the acceptability of care delivery.

Conclusions: The Forensic HCP/CSU relationship had an enabling focus to care delivery associated with a more positive experience for all and supported CSUs to live as independently as possible. Mapping this relationship against a conceptual framework provides an understanding of the CSU/HCP relationship characteristics that facilitates it to be enabling. Understanding implementation is key in being able identify the enablers and barriers to embedding shared care into everyday practice. Consideration of factors influencing adoption and dissemination of innovations in healthcare is important to inform the future strategy of shared care.

Strengths and limitations of this study:

- The CSU recruitment method required them to be invited to the research by their own clinical team and subsequently carers were to be invited by CSUs.
- Identification of potential participants relied on two information sources being up-to-date; the Denzapine® Monitoring Service website and the electronic medical notes.
- Aligned with IPA methodology, interviews and 'professionally' homogenous focus groups were used to collect the qualitative data.
- Quality assurance processes were completed to provide validity to this research, for example piloting topic guides, supervised focus groups, co-coding and re-coding of data.
- Data validity is reflected in the ability to substantiate the analysis with participant quotes and provide a dialogue of discussion between the results and existing literature.

For peer review only

Funding

This work was supported by a small research grant awarded by the College of Mental Health Pharmacy.

Competing Interests

All authors have completed the ICMJE uniform disclosure form at www.icmje.org/coi_disclosure.pdf and declare: Mrs. Sowerby reports grants from College of Mental Health Pharmacy (CMHP), during the conduct of the study. Dr. Taylor reports that at the time of the grant being awarded was a council member of the CMHP, but the award allocation was judged by a grant advisory panel with due process and alsocompleted two advisory boards for Sunovion (Lurasidone) during this time; and is a trustee for BathMind.

Data sharing

All data collected during this study has been included in the analysis and pubished.

Introduction

In recent years a number of United Kingdom (UK) government documents have been published concerning the provision of healthcare and mental health services, including: Equity and excellence: liberating the NHS[1], No Health without Mental Health[2] and the Five Year Forward View[3]. The main focus of these publications is there should be choice in how patients obtain their care, and care should be individualised and recovery focused to improve patients' independence.

Clozapine is the only antipsychotic with efficacy in treatment resistant schizophrenia (TRS) and is effective in around one-third of these patients[Error! Bookmark not defined.,5]. Due to the risk of clozapine-associated agranulocytosis, there is a requirement to undertake regular full blood count monitoring (FBC) and associated limitations on amount of medication supplied[6]. Additionally in the UK all prescribers, organisations dispensing clozapine and people taking clozapine, have to be registered with a brand specific clozapine website dependent on trust contract. An alternative model of clozapine provision to the usual clozapine clinic model is shared-care clozapine (see Table 1). In this model clozapine service users (CSUs) obtain their FBC monitoring, prescription and clozapine supply from their GP and community pharmacy respectively. In shared-care the responsibilities for clozapine provision are shared between specialist mental health and primary care healthcare professionals. Evidence suggests that shared-care clozapine is effective in maintaining the monitoring and management of clozapine in a less restrictive care pathway[7]. Studies have highlighted that shared-care clozapine is not suitable for all CSUs and propose criteria to identify those most suitable[7,8].

A growing body of evidence of the benefits of a shared-care approach in other long-term conditions including serious mental illness, is evolving[9,10]. Currently no published literature explores the perceptions and experiences of those that use and/or provide a shared-care clozapine service. The importance of patient experience of healthcare services is increasingly recognised in healthcare policy, to shape and inform service development, and payment structures for providers. Through understanding individuals' perceptions and experience of shared-care clozapine, insight can be gained into whether shared-care clozapine fulfils the government's vision for mental health care, and inform future service developments to improve quality and patient experience.

Aims of the study

For peer review only

1. To explore perceptions and experiences of people receiving and/or delivering a shared-care clozapine service

2. To gain an understanding of the effectiveness and acceptability of a shared-care clozapine service

Ethical approval

This study took place within one National Health Service (NHS) mental health trust in the UK and a favourable NHS ethics committee opinion, along with appropriate local research approval was received in 2013. (Reference 13/EM/0286).

Method

Semi-structured interviews and focus groups methods were used to explore perceptions and experiences of clozapine service users (CSUs), their carers, general practitioners (GPs); community psychiatric nurses (CPNs), social workers (SWs), community and hospital pharmacy staff and responsible clinicians (RCs) (See Table 2 for topic guide). The HCP topic guide was tested in a focus group whose pharmacist participants and all topics guides were amended as necessary. Topic guides were also reviewed after each interview or focus group if questions were found to be too direct, leading or closed. To be eligible for participation, healthcare (HCPs) needed to be involved with at least one CSU who obtained their clozapine through shared-care clozapine. CSUs were eligible to participate as long as they currently received their clozapine through shared-care and their lead consultant agreed they held capacity for consent. Carers of CSUs were eligible to participate if the CSU they cared for agreed to invite them to participate in the research.

Eligible CSUs, RCs and community pharmacists were identified via the Denzapine® Monitoring Service (DMS) website. GPs were identified from CSUs electronic notes by the researcher and hospital pharmacist participants were identified by the dispensary manager. All General Adult and Forensic community mental health teams (CMHT's), were targeted by the researcher to potentially recruit CPNs and SWs working in shared-care services, and potential CSU participants were approached by their care team. All potential participants received an information pack about the study, including an expression of interest form and given a minimum of two weeks to make their decision.

All interviews and focus groups were to be completed by CS and to be held at a convenient time and place for the participants, either in a room at their place of work or on a mental health trust site where participants could speak confidentially. Only researchers and participants were to be present during data collection and participant consent was to be received immediately prior to them taking part. Interviews and focus groups were audio recorded and transcribed verbatim to ensure a faithful reproduction of the participants' narratives and anonymised by removing identifiers. Transcripts were given an identifier e.g. Doctor 2, to enable comparison of the perceptions of the different HCP groups during the analysis stage.

Data analysis

As this research explored individuals' experiences and perceptions, interpretative phenomenological analysis (IPA) was used. In line with accepted analytical process for IPA each transcript was manually coded line-by-line into themes. Once super-ordinate or 'master' themes were identified for each participant group, the analysis moved to looking at connections and patterns across all participant groups. No software was used to aid the analysis of data or themes. The intention of IPA is to elicit meaning from participants' narratives that are interpreted by the researcher [11]. Robustness of the analytical process[12] was assessed by the coding of a transcript by both CS and DAT. There was a high level of agreement regarding the codes and so CS continued with the coding and theming process. At the end of the coding process CS and DAT met to discuss and clarify emerging themes.

For peer review only

Researchers' Personal characteristics

CS (MPharmS) was a clinical mental health pharmacist in forensic services and this study formed part of her postgraduate MSc Research project. She completed relevant theoretical and experiential training in qualitative research methods, particularly in interpretative phenomenological analysis. CS completed all interviews and focus groups, however was observed on two occasions by DAT for appropriateness and effectiveness.

DAT (PhD, MSc, MPharmS, FFRPS, MCMHP) was a senior lecturer and research supervisor for CS; providing training for qualitative research methods, analyses, informed consent and capacity assessment.

Relationship with participants

CS worked within the forensic service in the mental health trust where the study was conducted. Due to her role as clinical pharmacist within the forensic service, CS had developed professional relationships with some (but not all) of the primary and secondary care HCPs prior to the conduct of the study; she did not know any of the CSUs. All participants were aware this study was being undertaken as part of a further degree. The intention of IPA is to elicit meaning from participants' narratives that are interpreted by the researcher; thereby acknowledging the expertise of CS in shared-care clozapine whilst simultaneously preserving the voice of the participant through the provision of excerpts of participant narratives.

DAT knew none of the participants involved in the study, but had previously worked in and researched delivery of clozapine services. Reflexivity throughout the study enabled personal and professional bias to be recognised and addressed.

Results

A total of thirty-eight participants were recruited; including 32 healthcare professionals (HCPs) and 6 CSU's (General Adult CMHT = 1; Forensic = 5) and completion of 14 interviews and 6 'professionally' homogenous focus groups from General Adult and Forensic CMHT's; demographics of participants can be found in Table 3 All professional groups were similarly represented, except for carers as no expressions of interest to participate were received. No interviews or focus groups were repeated. Duration of interviews and focus groups ranged from 30 minutes to one and a half hours; none were repeated. Due to limitations in time and funding, transcripts and findings were not returned to participants for comment or correction. Field notes were taken for each interview or focus group to support reflexivity. Data saturation was achieved as no new themes emerged during the analysis of the final transcripts. The findings reported describe the participants experience and perceptions of shared-care clozapine as a service.

Four superordinate themes emerged from the data; The Sharing of Care, Multi-professional Relationships, The Provision of Care and the Clozapine Process. The inter-connectedness of these themes and minor themes is shown in Figure 1.

Excerpts from participant narratives (shown in indented italic text) have been chosen as examples of experiences and perceptions; these have been changed as little as possible to retain data authenticity. Names shown after each extract are fictitious to maintain participant anonymity.

Clozapine Process

Knowledge of the process of shared-care clozapine provision had a positive effect on the experience of shared care for most participants, as it frequently led to the development of a routine and greater confidence to engage with other members of the wider team. Learning by experience was the main

For peer review only

way participants gained new knowledge which in turn informed their understanding of the purpose of shared-care and what their role required of them.

> *"I guess I didn't understand all the ins and outs, I understand the monitoring and the reasons for the monitoring but I probably didn't understand all the things that could go wrong and how that…now I understand a lot more the things that happen that make it wrong. So like I said to you we have been learning as we've gone along what's required of us,"* Charles, Community Pharmacist

Stability and capability were used as surrogate markers by HCPs when deciding whether a CSU would be suitable for shared-care. With experience, Forensic CMHT HCPs realised that a CSU's current capability was not a true reflection of their full potential, and perceived that shared-care could be used to realise CSU's this potential. By acknowledging shared-care as part of the care pathway this ultimately led to a greater engagement with shared-care.

> *"[A CSU] as well who was really chaotic initially, and we were really worried about whether he'd manage it all, but it's actually made him get a lot more organised."* Bethany, Forensic CPN

In contrast, lack of familiarity and unclear rationale for shared-care limited the motivation of hospital pharmacy and General Adult CMHT HCPs, to proactively transfer CSUs over to shared-care.

The Sharing of Care

The sharing of care reverberated far wider than just the prescribing and monitoring of clozapine and was associated with its own obstacles, as it required individuals to work in a different way. Shared care shifted the responsibility for managing shared-care from secondary care to primary care, leading to anxiety and apprehension in both healthcare sectors.

> *"It's not a drug I'm totally at ease with, so there are apprehensions when any specialist says please do this, these are the protocols and unless you really feel you've got a backup, you feel somewhat exposed that hasn't particularly been an issue because the support has been there."* Dr White, GP

CSUs viewed shared-care as a supportive opportunity to develop their independence and take back ownership of their health, which in turn boosted their self-esteem. Over time Forensic CMHT HCPs saw that shared-care provided an opportunity to support CSUs to independence and enable them to realise their full capabilities by giving them back control. This process was facilitated by a change in the HCP/CSU relationship to one of upskilling CSUs, with CSUs being at the centre of shared-care, rather than the HCPs undertaking the process on their behalf. CSUs continued to drive the transition to independence and were often more engaged with their clozapine and physical health.

> *"Initially it was savings because….quite some time once I saw how the first person was working and did….once I saw the difference it made with him, how then we felt about him taking medication and how he was responding to that, I think then I moved away from seeing it as a cost saving exercise and seeing it as something that could actually useful in maintaining independence for the client."* Nick, Forensic SW

Commonly HCPs described assumptions, made either by themselves or others, which limited their trust in CSUs ability to take on an increased responsibility for managing their clozapine through shared care. General Adult CMHT HCPs highlighted that due to the way clozapine was prescribed and managed, it possessed a status that prevents CSUs from being discharged from the CMHT

regardless of their level of input. This in turn, influenced HCPs perception of the role of shared care in this patient group. Additionally many secondary care HCP's recognised that a greater level of CMHT involvement with the CSUs prevented their progression to independence, and restricted the development of a relationship with the GP.

> "*I suppose* (it) *depends how well they are doesn't it, how insightful they are in the first place, how sassy they are with how systems work and stuff really at the end of the day, they just want their meds to turn up when they need more (CPN) comes round at 4 with the meds, that's what they want isn't it?*" *Jane, General Adult CPN*

The Provision of Care

Providing care to people with psychosis included not only clozapine as a treatment, but also the role each member of the MDT played to support the recovery and management of the individual. The Forensic CMHT demonstrated a more enabling and recovery focused provision of care, compared to the more paternalistic style exhibited by the General Adult CMHT. The Forensic CMHT perceived that their role was to monitor and assist CSUs to live as independently as possible by recognising them as a contributory member of the team. Together CSUs were supported into a routine and educated on the processes of the shared-care system. In contrast, the General Adult CMHT retained all responsibility for the process, perceiving that their role was to ensure that everything went right with the supply of clozapine as they didn't want to cause any additional problems for CSUs.

> "*Difficult I would say, very difficult, lots of chasing finding about results, seeing if the pharmacy understands you and knows what you're trying to ask for, and then trying to establish whether they've actually got the medication or not, when they're going to get it then you've got to faff around, pick it up and get it to the patient it's a nightmare.*" *Kate, General Adult CPN*

The General Adult RC's felt they were overburdened with their workload and struggled to find time to understand shared-care and therefore their role within it. Conversely, the Forensic RCs were clearer as to their role of monitoring CSUs in the community and making any necessary changes to clozapine treatment via the GP. This enabled them to redistribute their time to tasks requiring greater specialist knowledge. GPs perceived their main role in shared-care to be prescribing clozapine and checking FBC results, however they also felt a large part of their role was liaison, including with the secondary care team regarding any CSU or clozapine-related problems.

> "I *suppose my, I think I see patients regularly as do the rest of the clinical team and I suppose we're keeping an eye on them to make sure that that person is still taking their clozapine they're still happy with it obviously if there are other issues that might need dose changes or whatever then we're involved in doing that." Dr Taylor, Forensic RC*

CSUs perceived their role in shared-care was to attend the GP surgery for an FBC, ordering their prescription, and collecting and taking their clozapine. They found HCPs in primary and secondary care were a source of support when needed, and valued the additional services offered by community pharmacy in helping them fulfil their own role. Participating in shared-care clozapine was often seen as rewarding and satisfying; acting as an internal motivator for both HCPs and CSUs to continue engaging with shared care.

For peer review only

"Gives us the onus and responsibility, which is good in the sense that it's a responsibility that we've got to take on and I think it helps rather than hinder." Adam, CSU

In practice, community pharmacists often found that they were heavily involved in identifying the missing elements of the process which prevented them undertaking their clozapine dispensing role, whereas hospital pharmacy perceived their main role to be transferring CSUs to shared-care when requested by another HCP. This would involve co-ordinating the process and communicating with the various HCPs involved in both primary and secondary care.

"A lot of the work is the setting up of a new patient. I think it's getting all that co-ordinated, getting confirmation from the consultant, the GP, the Chemist, the patient, co-ordinating that and then when that's all through liaising with Denzapine to get them registered for the shared care and then it's that, I think it's mainly the co-ordinating of it all at the beginning of the stage." Emily, Hospital pharmacy

Clozapine was perceived by all HCPs and CSUs to be significantly different to other medicines because of the supply and monitoring requirements. Not only did HCPs feel they were more involved with CSUs and more reliant on other members of the MDT, including CSUs, but also system processes to a greater extent compared to individuals on other antipsychotics. Despite this many healthcare professionals and all CSUs thought that clozapine often provided the best opportunity for recovery and hospital discharge in individuals with schizophrenia; particularly if they had already tried other antipsychotics with little success. These highly valued benefits of clozapine provided the motivation for CSUs to continue taking it despite the negatives associated with clozapine treatment.

"The other thing with Clozapine which was explained to me is side effects and pluses and minuses and sort of thing, but it's like to me it's taken something that keeps me well. I class it the same as eating food and drinking water. I need that to keep me level and keep me… I feel totally normal now whereas I couldn't before and I was on all of the old medications [….] whole range of different and they never really worked. Clozapine was the first one that ever worked the only downside to it was the side effects the dribble, makes you sleep, makes you weight gain, things like that but when you weigh up the good and the bad Clozapine is up there it does the job." Adam, CSU

Multi-professional Relationships

Being recognised as a member of a team supported the development of multi-professional relationships and communication. Relationships were integral to building trust and understanding each others' role and expertise. Knowing other team members had a positive influence on communication, as CSUs and HCPs were more comfortable to make contact with someone if they knew them. Forensic CPNs and SWs described how their relationships and communication with hospital and community pharmacists and GPs had improved with working together in shared-care. The building of trust and understanding that occurs in a relationship informed the support offered by the relationship to HCPs and CSUs.

"Knowing that there is somewhere that you can call on is important and certainly the particular community mental health nurse who is involved in the care she is excellent. Apart from these meetings she is available for discussion and she has

For peer review only

come along and organised blood tests and like if there seems to be a more urgent situation where some compliance has gone wrong." Dr White, GP

"I think if there was a problem with one of my guys in the (community) *pharmacy where I've been in I'll actually go in and talk to him* (community pharmacist) *now whereas before I'd come through* (the hospital pharmacy) *so I talk to him now*." *John, Forensic CPN*

Sharing responsibility through shared-care was seen as a source of support for primary care by GPs and RCs, as it reinforced a sense of an equal and shared responsibility of the team in ensuring the provision of clozapine to CSUs.

"Well it enables us to umm to manage this difficult client group for patients that need to take it, so we are happy to accept our share of the responsibility for that and…. so it is a positive thing and it helps us with our sort of multi-disciplinary work." Dr Hudson, GP

Discussion

Principle findings

All participants perceived clozapine provision as different, because of the process of supply and its beneficial effects on symptoms, but also the negative effects of treatment. Having a good understanding of the processes involved had a positive effect on an individual's experience of shared-care clozapine, as they possessed greater confidence to engage and integrate it within the patient pathway. The main way of gaining understanding, not only with the processes but also of what could be achieved by integrating this service within the patient pathway, was through having experience of shared-care clozapine. Over time the routine use of shared-care supported the development of multi-professional relationships with HCPs and CSUs; this led to improved communication and enabled HCPs to feel valued and included within the MDT. The use of a shared-care service reinforced a sense of equal and shared responsibility for RCs and GPs, further supporting the development of multi-professional relationships and an additional sense of support. These relationships were integral to building trust and understanding of each other's role and expertise, which was essential to continuing to build confidence and engagement with shared-care clozapine.

CSU stability and capability were used as surrogate markers by HCPs for identifying those most suitable for shared-care clozapine. Despite clozapine being potentially suitable, some HCPs held assumptions about CSUs that influenced their ability to trust them. For the General Adult CMHT this reluctance to relinquish a degree of responsibility to CSUs led to a paternalistic CSU/HCP relationship. In contrast the Forensic CMHT recognised by changing their relationship with CSUs to an enabling one, shared-care clozapine provided an opportunity to further assist CSU's take back ownership of their health and develop independence through realisation of their capability. This provides insight into the highly apparent variation in the implementation of shared-care clozapine between the General Adult CMHT and the Forensic CMHT. For all participant CSUs, shared care was rewarding and satisfying; acting as an internal motivator for both HCPs and CSUs to continue engaging with shared care.

Implications for Practice

The enabling and recovery focus to care in the Forensic HCP/CSU relationship was not only associated with a more positive experience for HCPs and CSUs, but also a more consistent

For peer review only - http://bmjopen.bmj.com/site/about/guidelines.xhtml

implementation and integration of shared-care into the patient pathway. This enabled the Forensic CMHT to support CSUs to live as independently as possible and enable them to realise their full capabilities by giving them back control. The Forensic HCP/CSU relationship appears to possess a more symmetric, co-operative, intimate and social characteristic if mapped onto the Wish conceptual model of relationships[13]. This model demonstrates the interplay of power (centeredness), valence (nature), intensity (commitment), and formality (affect) as demonstrated in Table 4 (adapted from Closing the Gap through Changing Relationships: evaluation [15]) .

This characterisation of relationship is apparent as the care provided by the Forensic CMHT seems to be centred around the CSU rather than the HCP. The Forensic CMHT share an aim of greater independence and normalisation for their CSUs, demonstrating commitment and willingness to adapt their beliefs and behaviours, and involve primary care HCPs to a greater degree to support the CSU in developing greater independence.

The characterisation of the Forensic HCP/CSU could be said to fulfil all of the conditions of the enablement principle of person-centred care suggested by The Health Foundation[14,15]. This suggests that shared-care clozapine could not only enhance CSU independence and integration into primary care but also enhance the provision of person-centred care, therefore fulfilling the government's ambition of the routine provision of person-centred care[1-3]. When providing a service such as shared-care clozapine, the HCP/CSU relationship needs to be considered as this can not only influence individual's experience, but also perception of the worth of the service.

The implementation of shared-care clozapine services ideally requires a change in HCP practice and attitude/perspective, to achieve the maximum and most positive impact for CSUs and requires consideration of a number of points. HCPs, either individually or collectively, undertake 'sense-making work' when faced with the challenge of implementing a set of practices within a specific context (for example shared-care). Therefore, it is important to ensure that the rational and perceived advantages of implementing a new service are clearly aligned with staff priorities. Quantifying what will be required of them and how this differs to the current situation is important as it highlights compatibility with their existing skills and knowledge. Maintaining simplicity will also support buy-in and therefore implementation.

This 'relational work' is required to engage and secure commitment of individuals to execute the new service. Initiating a shared-care clozapine service with a small, select group of CSUs provides an advantage, as trialability supports the spreading and adoption of innovation. Clarifying risk management and committed support from the wider team during this time encourages individuals to participate in this initial implementation phase. Subsequently when trialling shared-care clozapine it is valuable to provide individuals an opportunity to assess and understand the way the new service affects themselves and others. This evaluation can subsequently be used to amend perceived advantages and rationale of the service and the individuals' or teams' perceived compatibility with the skills and knowledge required. Thus potentially identifying additional required support or training to sustain individual's engagement and adoption of shared-care into their practice. The impact of cultural and organisational factors should also be considered when implementing a change such as shared-care clozapine.

<u>Strengths and limitations</u>

This is the first qualitative study to explore the experiences and perceptions of those that use and provide a shared-care clozapine service.

Aligned with IPA methodology, interviews and 'professionally' homogenous focus groups were used to collect qualitative data. Quality assurance processes were completed to provide validity to this research for example piloting topic guides, supervised focus groups, co-coding and re-coding of data.

For peer review only

Data validity is reflected in the ability to substantiate the analysis with participant quotes and provide a dialogue of discussion between the results and existing literature.

In terms of group size, CSUs were not equally represented in number, there were more CSUs from the Forensic CMHT compared to General Adult CMHT. This may mean the experiences and perceptions of CSUs from General Adult CMHT may not be sufficiently represented. The CSU recruitment method required them to be invited to the research by their own clinical team. The greater recruitment of CSUs from the Forensic CMHT could be related to their enabling approach to care or a reflection of the team's experience of shared-care in comparison to General Adult CMHT.

Acknowledgements

This study was funded by a small grant from the College of Mental Health Pharmacy. We thank all study participants for their input into this study and for the team members who helped in identification of potential participants.

Author Contribution

Camilla Sowerby

- Substantial contributions to the conception, design; and acquisition, analysis, and interpretation of data for the work; AND
- Drafting and critically revising the work; AND
- Final approval of the version to be published; AND
- Agreement to be accountable for all aspects of the work in ensuring that questions related to the accuracy or integrity of any part of the work are appropriately investigated and resolved.

Denise Taylor

- Substantial contributions to the conception, design, analysis and interpretation of data of the work; AND
- Critical revision of the work; AND
- Final approval of the version to be published; AND
- Agreement to be accountable for all aspects of the work in ensuring that questions related to the accuracy or integrity of any part of the work are appropriately investigated and resolved.

References

1 DH. *Equity and excellence: Liberating the NHS*. July 2010.
2 HMG/DH. *No Health Without Mental Health: A Cross-Government Mental Health Outcomes Strategy for People of All Ages*. February 2011.
3 NHS England. *Five Year Forward View*. October 2014.
4 Cowen P, Harrison P, Burns T. *Shorter Oxford Textbook of Psychiatry* (Chapter 11, Schizophrenia). Oxford University Press, 2012.
5 Kane J, Honigfeld G, Singer J, Meltzer H, The Clozaril Collaborative Study Group. Clozapine for the treatment-resistant schizophrenic: results of a US multicentre trial. *Psychopharmacology*. 1989; **99**: S60-S63.
6 Britannia Pharmaceuticals. *Denzapine 100mg tablets - Summary of Product Characteristics* (15th January 2015). Accessed via: http://www.medicines.org.uk/emc/medicine/25965.

For peer review only

7 Filia S, Lee S, Sinclair K, Wheelhouse A, de Castella A et al. Demonstrating the effectiveness of less restrictive care pathways for the management of patients treated with clozapine. *Australasian Psychiatry*. 2013; **21(5)**: 449-455.

8 Filia S, Wheelhouse A, Lee S, Main M, de Castella A, Wilkins S et al. Transitioning patients taking clozapine from the public to private/GP shared-care setting: Barriers and criteria. *Australian and New Zealand Journal of Psychiatry*. 2012; **46(3)**: 225-231.

9 Kelly B, Perkins D, Fuller, Parker S. Shared care in mental illness: A rapid review to inform implementation. *International Journal of Mental Health Systems*. 2011; **5**: 31.

10 Smith SM, Allwright S, O'Dowd T. Effectiveness of shared care across the interface between primary and speciality are in chronic disease management. *Cochrane Database of Systematic Reviews*. 2007.

11 Smith J, Flowers P, Larkin M. *Interpretative phenomenological analysis: Theory, Method and Research*. Sage, 2013.

12 Lincoln YS, Guba EG. *Naturalistic enquiry*. Sage, 1985.

13 Wish M. Comparisons among multidimensional structures of interpersonal relations. *Multivariate Behav Res*. 1976; **11(3)**: 297-324.

14 The Health Foundation. *Person-centred care made simple*. 2014.

15 The Health Foundation. *Closing the Gap through Changing Relationships: evaluation*. 2014.

For peer review only

Table 1: Description of models used to provide clozapine

	**Clozapine clinic**	**Shared care**
FBC monitoring	CSUs attend the clinic, which is held at a hospital trust site, where FBC monitoring is undertaken and sent to the clozapine company for processing.	FBC monitoring is undertaken by GPs and either sent to the local pathology laboratory or posted to the clozapine company for processing.
Prescription	Same as CMHT-based supply.	The GP prescribes clozapine on an FP10 prescription. The CSU maintains regular out-patient appointments with their consultant. The frequency of these depend on the CSU. CSUs can only use the shared care service if they have been on clozapine for 12 months and are stabilised on treatment.
Dispensing	Hospital pharmacy dispenses clozapine to the clozapine clinic. CSUs collect their clozapine from the clinic.	Clozapine is supplied against the FP10 by the community pharmacy the CSU wishes to use. The community pharmacy needs to be registered with the clozapine company in order to be able to supply clozapine. CSUs either collect their prescription from the community pharmacy or utilise their delivery services.

For peer review only

Table 2: Focus group and interview topic guides

CSU topic guide	
Questions	Probing questions
When you were asked about participating in the shared care clozapine service, what were your first thoughts?	Why did you agree to participate in the clozapine shared care service?<br>How did it make you feel?<br>What does the clozapine shared care service mean to you?
What is your experience of being involved in the shared care clozapine service?	Describe the differences to you by obtaining your clozapine through the shared care service<br>Can you identify positive and negative influences on your experience?<br>How does that make you feel?<br>What does that mean to you?<br>How has the shared care clozapine service impacted on you?<br>How have your thoughts and feelings towards the clozapine shared care service changed over time?
Describe the roles of the healthcare professionals that provide the clozapine shared care service.	How has the clozapine shared care service affected your thoughts and feelings about the healthcare professionals providing the clozapine shared care service?<br>Has the shared care service changed your relationship with these professionals?<br>In what way has the clozapine shared care service affected thoughts and feelings about these healthcare professionals?

For peer review only

HCP topic guide	
Questions	Probing questions
What is your experience of being involved in the shared care clozapine service?	What were your first thoughts and feelings when you were asked to participate?<br>Can you identify positive and negative influences on your experience?<br>How does that make you feel?<br>What does that mean to you?<br>How has the shared care clozapine service impacted on you?
What do you think the differences are in care for someone if they receive their clozapine through the shared care clozapine service compared to other means e.g. secondary care?	What do you think is the benefits are?<br>What do you think the negatives are?<br>What do you think was trying to be achieved with the shared care agreement?<br>What do you think is actually achieved with the shared care service?<br>What does this mean for you?
Can you describe your role within the shared care clozapine service?	What does your input mean to you?<br>How does it make you feel?<br>How has the shared care clozapine service affected your working relationship with other professionals?<br>What does this mean for you?<br>How has the shared care clozapine service developed you as a professional?<br>What training/support have you received?/how have you received this training?<br>What other ways would you like to get involved more in the care of these patients?

Table 3: Demographics of focus group participants

Focus groups			
**Participants***	**Profession**	**Sector**	**Years with shared-care clozapine**
Mark<br>Emily<br>Laura<br>Angela	Hospital Pharmacist<br>MMT¥<br>MMT¥<br>Receptionist	Hospital Pharmacy, Secondary care	1.5<br>2<br>5<br>5
Luke<br>Bethany<br>John	CPN<br>CPN<br>CPN	Forensic CMHT, secondary care	2<br>2<br>2
Kate<br>Anne<br>Denise	CPN<br>CPN<br>CPN	General Adult CMHT, secondary care	4<br>4<br>4
Jennifer<br>Trace<br>Victoria	CPN<br>SW<br>CPN	General Adult CMHT, secondary care	2<br>5<br>1.5
Lucy<br>Jane<br>Nick<br>Claire	SW<br>SW<br>SW<br>SW	Forensic CMHT, secondary care	2<br>2<br>2<br>2

For peer review only

Tim<br>Simon<br>Adam<br>Frank<br>Rob	CSU<br>CSU<br>CSU<br>CSU<br>CSU	Attached to the Forensic CMHT	1-2<br>1-2<br>1-2<br>1-2<br>1-2

* Pseudonym names used for confidentiality ¥ Medicines Management Technician

For peer review only

Table 4: Wish's conceptual model of relationships

Power	Valance	Intensity	Formality
Symmetric ↔ Asymmetric	Cooperative ↔ Competive	Intimate ↔ Distant	Social ↔ Professional
Agency<br>Dependence<br>Centredness	Communication<br>Agreement<br>Conflict<br>Agenda<br>Trust	Commitment<br>Transactional/<br>relational	Affect<br>Exclusivity

*adapted from Closing the Gap through Changing Relationships: evaluation [15]

For peer review only

Figure 1: Illustration of superordinate themes and their connections

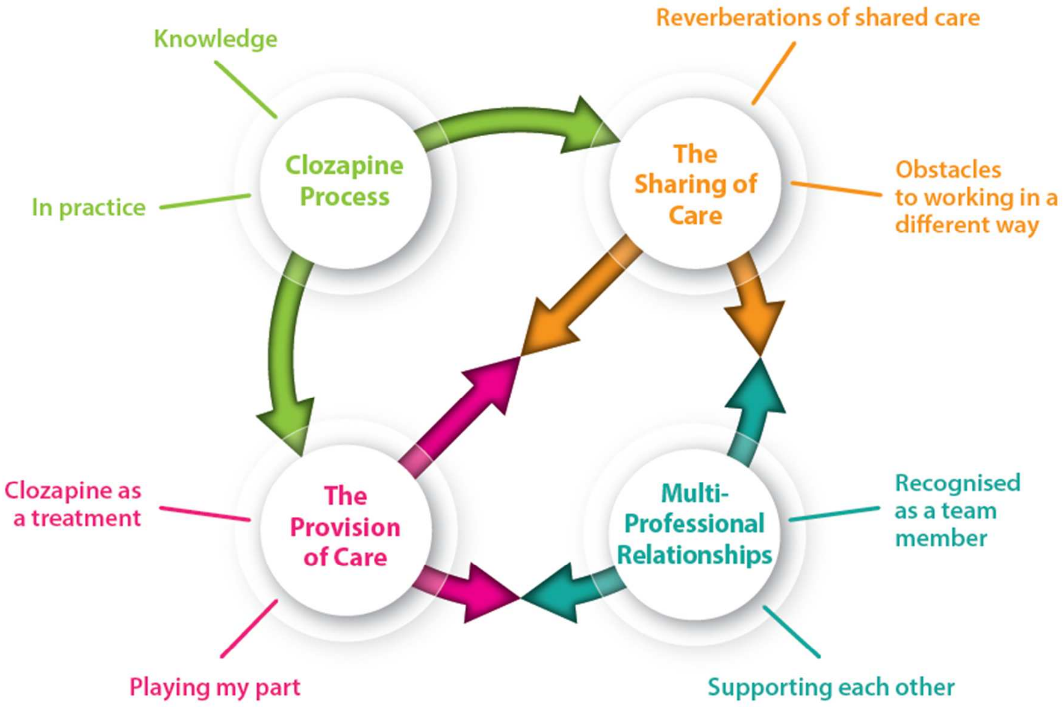

For peer review only

COREQ:

Domain One: Research team and reflexivity

**No.**	**Item**	**Guide questions/description**	**In Paper**
**Personal Characteristics**			
**1**	Interviewer/Facilitator	Which author conducted IV/FGs?	Yes
**2**	Credentials	What were the researcher's credentials? *E.g. PhD, MD*	Yes
**3**	Occupation	What was their occupation at the time of the study	Yes
**4**	Gender	Was the researcher male or female?	Yes
**5**	Experience & training	What experience or training did the researcher have?	Yes
Relationship with participants			
**6**	Relationship established	Was a relationship established prior to study commencement?	Yes
**7**	Participant knowledge of the interviewer	What did the participants know about the researcher? e.*g. personal goals, reasons for doing the research*	Yes
**8**	Interviewer characteristics	What characteristics were reported about the interviewer/facilitator? e.g. *Bias, assumptions, reasons and interests in the research topic*	Yes

Domain Two: Study Design

**No.**	**Item**	**Guide questions/description**	**In Paper**
Theoretical framework			
**9**	Methodological orientation and Theory	What methodological orientation was stated to underpin	yes

For peer review only

		the study? *e.g. grounded theory, discourse analysis, ethnography, phenomenology, content analysis*	
Participant selection			
**10**	Sampling	How were participants selected? *e.g. purposive, convenience, consecutive, snowball*	yes
**11**	Method of approach	How were participants approached? e.*g. face-to-face, telephone, mail, email*	yes
**12**	Sample size	How many participants were in the study?	yes
**13**	Non-participation	How many people refused to participate or dropped out? Reasons?	Yes
**Setting**			
**14**	Setting of data collection	Where was the data collected? e.*g. home, clinic, workplace*	Yes
**15**	Presence of non-participants	Was anyone else present besides the participants and researchers?	Yes
**16**	Description of sample	What are the important characteristics of the sample? *e.g. demographic data, date*	Yes
**Data Collection**			
**17**	Interview guide	Were questions, prompts, guides provided by the	Yes

For peer review only

		authors? Was it pilot tested?	
**18**	Repeat interviews	Were repeat interviews carried out? If yes, how many?	Yes
**19**	Audio/visual recording	Did the research use audio or visual recording to collect the data?	Yes
**20**	Field notes	Were field notes made during and/or after the interview or focus group?	Yes
**21**	Duration	What was the duration of the interviews or focus group?	Yes
**22**	Data saturation	Was data saturation discussed?	Yes
**23**	Transcripts returned	Were transcripts returned to participants for comment and/or correction?	Yes

Domain 3: Analysis and Findings

**No.**	**Item**	**Guide questions/description**	**In Paper**
**Data analysis**			
**24**	Number of data coders	How many data coders coded the data?	Yes
**25**	Description of the coding tree	Did authors provide a description of the coding tree?	Yes
**26**	Derivation of themes	Were themes identified in advance or derived from the data?	Yes
**27**	Software	What software, if applicable, was used to manage the data?	Yes
**28**	Participant checking	Did participants	Yes

For peer review only

		provide feedback on the findings?	
**Participant Checking**			
**29**	Were participant quotations presented to illustrate the themes / findings?	Were participant quotations presented to illustrate the themes / findings? Was each quotation identified? e.*g. participant number*	Yes
**30**	Data and findings consistent	Was there consistency between the data presented and the findings?	Yes
**31**	Clarity of major themes	Were major themes clearly presented in the findings?	Yes
**32**	. Clarity of minor themes	Is there a description of diverse cases or discussion of minor themes?	Yes

For peer review only

BMJ Open

Cross Sector User and Provider Perceptions on Experiences of Shared Care Clozapine – a qualitative study

Journal:	*BMJ Open*
Manuscript ID	bmjopen-2017-017183.R1
Article Type:	Research
Date Submitted by the Author:	17-Jul-2017
Complete List of Authors:	Sowerby, Camilla; Universtiy of Bath, Pharmacy and Pharmacology<br>Taylor, Denise; Universtiy of Bath, Pharmacy and Pharmacology
**Primary Subject Heading**:	Mental health
Secondary Subject Heading:	Qualitative research, Patient-centred medicine, Health services research, General practice / Family practice
Keywords:	MENTAL HEALTH, QUALITATIVE RESEARCH, Schizophrenia & psychotic disorders < PSYCHIATRY

SCHOLARONE™
Manuscripts

For peer review only

For peer review only - http://bmjopen.bmj.com/site/about/guidelines.xhtml

TITLE PAGE

Title: Cross Sector User and Provider Perceptions on Experiences of Shared Care Clozapine – a qualitative study

Mrs Camilla Sowerby and Dr Denise Taylor

Corresponding author: Mrs Camilla Sowerby, Early Intervention in Psychosis Pharmacist, The Old School House School Lane Aston Rowant, OX49 5SU, Camilla.sowerby@berkshire.nhs.uk, 07876216828

Co-author: Dr Denise Taylor, Senior Lecturer, Department of Pharmacy and Pharmacology, University of Bath, Bath, UK. D.A.Taylor@bath.ac.uk

Key words: Shared-care, Clozapine service, Primary care, Cross sector working, Multi-professional working

Word count 5772 words

Author Contribution

Camilla Sowerby and Denise Taylor

- Substantial contributions to the conception, design; and acquisition, analysis, and interpretation of data for the work; AND
- Drafting and critically revising the work; AND
- Final approval of the version to be published; AND
- Agreement to be accountable for all aspects of the work in ensuring that questions related to the accuracy or integrity of any part of the work are appropriately investigated and resolved.

Competing Interests

Both authors have completed the ICMJE uniform disclosure form at www.icmje.org/coi_disclosure.pdf and declare: Mrs. Sowerby reports grants from College of Mental Health Pharmacy (CMHP), during the conduct of the study. Dr. Taylor reports that at the time of the grant being awarded was a council member of the CMHP, but the award allocation was judged by a grant advisory panel with due process and also completed two advisory boards for Sunovion (Lurasidone) during this time; and is a trustee for Bath Mind.

Funding

This work was supported by a small research grant awarded by the College of Mental Health Pharmacy.

Data sharing

All data collected during this study has been included in the analysis and is available for secondary analysis on request.

For peer review only

ABSTRACT (n= 291)

Objectives: To explore individual perceptions on experiences of people receiving and/or delivering a shared-care clozapine service.

Design: Interpretative phenomenological analysis guided the delivery and analysis of a semi-structured interview and focus group study designed to explore participant experience of shared care clozapine. Ethical approval 13/EM/0286 was gained in July 2013 from East Midlands – Nottingham 1 REC.

Participants: Eight stakeholder groups from Adult and Forensic Mental Health involved in shared care clozapine provision delivered in primary care, were identified for recruitment from one mental health trust in England (six different groups of healthcare professionals (HCPs), clozapine service users (CSUs) and their carers. To be eligible for recruitment all potential participants had to be either providing, receiving or the carer of a person receiving clozapine by shared care.

Results: 32 HCPs and 6 CSUs were recruited and 14 interviews and 6 participant homogenous focus groups were run. Four shared superordinate themes were identified; Clozapine Process, The Sharing of Care, The Provision of Care and Multi-professional Relationships. Differences between Adult and Forensic engagement in shared care were noted and both HCP-CSU relationship were mapped to the Wish conceptual framework of relationships to provide insight into how shared care clozapine can provide a mechanism for provision of person-centred care, which was present in the Forensic HCP-CSU but not General Adult HCP-CSU relationship.

Conclusions: The Forensic HCP/CSU relationship demonstrated how cross-sector working through shared care clozapine can provide a mechanism for provision of person-centred care by enabling a person-centred focus to care delivery which supported CSUs to live as independently as possible. Person-centred care demonstrably improves patient care outcomes and wider implementation of shared care clozapine could provide greater integration of people with serious mental illness and reduce stigma within the community whilst improving patient outcomes.

Strengths and limitations of this study:

- This is the first qualitative study to explore the experiences and perceptions of those that use and provide a shared-care clozapine service.
- Identification of potential participants relied on two information sources being up-to-date; the Denzapine® Monitoring Service website and the electronic medical notes.
- In terms of group size, CSUs were not equally represented in number, there were more Forensic CMHT CSUs compared to General Adult CMHT CSUs. This may mean the experiences and perceptions of CSUs from General Adult CMHT may not be sufficiently represented. The CSU recruitment method required them to be invited to the research by their own clinical team. The greater recruitment of CSUs from the Forensic CMHT could be related to their enabling approach to care or a reflection of the team's experience of shared-care in comparison to General Adult CMHT.
- No carers of CSUs participated, reflecting the isolation from family and loved ones that some people with serious mental illness live with.
- Data validity is reflected in the ability to substantiate the analysis with participant quotes and provide a dialogue of discussion between the results and existing literature.

For peer review only

INTRODUCTION (n=5578 – not including tables)

In recent years a number of United Kingdom (UK) government documents have been published concerning the provision of healthcare and mental health services, including: Equity and excellence: liberating the NHS,[1] No Health without Mental Health[2] and the Five Year Forward View.[3] The main focus of these publications is there should be choice in how patients obtain their care, and care should be individualised and recovery focused to improve patients' independence.

Clozapine is the only antipsychotic with efficacy in treatment resistant schizophrenia (TRS) and is effective in around one-third of these patients.**Error! Bookmark not defined.**[,5] Additionally in the United Kingdom, due to the risk of clozapine-associated agranulocytosis, there is a requirement to undertake regular full blood count monitoring (FBC) and associated limitations on amount of medication supplied.[6] Additionally in the UK all prescribers, organisations dispensing clozapine and people taking clozapine, have to be registered with a brand specific clozapine website dependent on trust contract. An alternative model of clozapine provision to the usual clozapine clinic model or community mental health team (CMHT) based supply is shared-care clozapine (see Table 1).

Table 1: Description of models used to provide clozapine

	**Community Mental Health Team (CMHT) based supply**	**Clozapine clinic**	**Shared care**
FBC monitoring	The FBC monitoring is undertaken by GPs and either sent to the local pathology laboratory or posted to the clozapine company for processing. Results are automatically uploaded onto the DMS website if sent to them for processing, whilst hospital pharmacy upload those results of FBC's sent to the local pathology laboratory.	Clozapine patients attend the clinic, which is held at a hospital site, where FBC monitoring is undertaken and sent to the clozapine company for processing.	The FBC monitoring is undertaken by GPs and either sent to the local pathology laboratory or posted to the clozapine company for processing.
Prescription	The prescriptions are managed by hospital pharmacy and written by the Responsible Clinician of the clozapine patient. The patient maintains regular out-patient appointments with their RC. The frequency of these depend on the individual patient.	Same as CMHT-based supply.	The GP prescribed clozapine on an FP10 prescription. The CSU maintains regular out-patient appointments with their RC. The frequency of these depend on the Clozapine Service User (CSU). CSUs can only use the shared care service if they have been on clozapine for 12 months and are stabilised on treatment.
Dispensing	Hospital pharmacy dispenses clozapine to the relevant CMHT site or posts the medicine to the patient. Patients either collect their prescription from the CMHT site or CMHT staff to deliver to them.	Hospital pharmacy dispenses clozapine to the clozapine clinic. Patients collect their clozapine from the clinic.	Clozapine is supplied against the FP10 by the community pharmacy the CSU wishes to use. The community pharmacy needs to be registered with the clozapine company in order to be able to supply clozapine. CSUs either collect their prescription from the community pharmacy or

For peer review only

			utilise their delivery services.

METHODOLOGY AND METHODS

In this model clozapine service users (CSUs) obtain their FBC monitoring, prescription and clozapine supply from their GP and community pharmacy respectively. In shared-care the responsibilities for clozapine provision are shared between specialist mental health and primary care healthcare professionals. Evidence suggests that shared-care clozapine is effective in maintaining the monitoring and management of clozapine in a less restrictive care pathway.[7] Studies have highlighted that shared-care clozapine is not suitable for all CSUs and propose criteria to identify those most suitable.[7,8]

A growing body of evidence of the benefits of a shared-care approach in other long-term conditions including serious mental illness, is evolving.[9,10] Currently no published literature explores the perceptions of experiences of those that use and/or provide a shared-care clozapine service. The importance of patient experience of healthcare services is increasingly recognised in healthcare policy, to shape and inform service development, and payment structures for providers. Through understanding individuals' perceptions and experience of shared-care clozapine, insight can be gained into whether shared-care clozapine fulfils the government's vision for mental health care, and inform future service developments to improve quality and patient experience.

Aims of the study

1. To explore individual perceptions on experiences of people receiving and/or delivering a shared-care clozapine service.
2. To gain an understanding of effectiveness and acceptability of shared-care clozapine.

Ethical approval

This study took place within one National Health Service (NHS) mental health trust in the UK and a favourable NHS ethics committee opinion (East Midlands – Nottingham 1 NRES: Reference 13/EM/0286), and local Research and Development approval was received in 2013.

Study Design

Interpretative Phenomenological Analyses (IPA) was selected as the method as it attempts to explore the personal experience as it is perceived by the participant, rather than the researcher producing an objective statement of the event itself. IPA also allows for the expertise of the researcher to be acknowledged by reflexivity in the analytical processes.[11] Semi-structured interviews and focus groups methods were used to explore perceptions and experiences of clozapine service users (CSUs), their carers, general practitioners (GPs); community psychiatric nurses (CPNs), social workers (SWs), community and hospital pharmacy staff and responsible clinicians (RCs) (See Table 2 for topic guide).

For peer review only

Table 2: Focus group and interview topic guides

**Clozapine Service User Topic Guide**		**Healthcare Professional Topic Guide**	
Questions	Probing questions	Questions	Probing questions
When you were asked about participating in the shared care clozapine service, what were your first thoughts?	Why did you agree to participate in the clozapine shared care service?<br>How did it make you feel?<br>What does the clozapine shared care service mean to you?	What do you think the differences are in care for someone if they receive their clozapine through the shared care clozapine service compared to other means e.g. secondary care?	What do you think is the benefits and negatives are?<br>What do you think was trying to be achieved with the shared care agreement?<br>What do you think is actually achieved with the shared care service?<br>What does this mean for you?
What is your experience of being involved in the shared care clozapine service?	Describe the differences to you by obtaining your clozapine through the shared care service.<br>Can you identify positive and negative influences on your experience?<br>How does that make you feel?<br>What does that mean to you?<br>How has the shared care clozapine service impacted you?<br>How have your thoughts and feelings towards the clozapine shared care service changed over time?	What is your experience of being involved in the shared care clozapine service?	What were your first thoughts and feelings when you were asked to participate?<br>Can you identify positive and negative influences on your experience?<br>How does that make you feel?<br>What does that mean to you?<br>How has the shared care clozapine service impacted on you?
Describe the roles of the healthcare professionals that provide the clozapine shared care service.	How has the clozapine shared care service affected your thoughts and feelings about the healthcare professionals providing the clozapine shared care service?<br>Has the shared care service changed your relationship with these professionals?<br>In what way has the clozapine shared care service affected thoughts and feelings about these healthcare professionals?	Can you describe your role within the shared care clozapine service?	What does your input mean to you? How does it make you feel?<br>How has the shared care clozapine service affected your working relationship with other professionals?<br>What does this mean for you?<br>How has the shared care clozapine service developed you as a professional?<br>What training/support have you received? How did you receive this training?<br>What other ways would you like to get more involved in the care of these patients?

For peer review only

For peer review only - http://bmjopen.bmj.com/site/about/guidelines.xhtml

The HCP topic guide was piloted with pharmacist focus group participants and amended following feedback. Topic guides were also reviewed after each interview or focus group if questions were found to be too direct, leading or closed. To be eligible for recruitment all potential participants had to be either providing, receiving or the carer of a person receiving, clozapine by shared care. Carers of CSUs were only eligible for recruitment if the CSU they cared for invited them to participate in the research.

Eligible CSUs, RCs and community pharmacists were identified via the Denzapine® Monitoring Service (DMS) website. GPs were identified from CSUs electronic notes by the researcher (CS) and hospital pharmacist participants were identified by the dispensary manager. All General Adult and Forensic community mental health teams (CMHT's), were targeted by the researcher to potentially recruit CPNs and SWs working in shared-care services, and potential CSU participants were approached by their care team. All potential participants received an information pack about the study, including an expression of interest form and given a minimum of two weeks to make their decision.

All interviews and focus groups were to be completed by Camilla Sowerby (CS) and held at a convenient time and place for participants, either in a room at their place of work or on a mental health trust site, where participants could speak confidentially. Only researchers and participants were to be present during data collection and participant consent was to be received immediately prior to them taking part. Each participant received a gift voucher as a token of appreciation for their time and input into the study. Interviews and focus groups were audio recorded and transcribed verbatim to ensure a faithful reproduction of the participants' participation and anonymised by removing identifiers. Transcripts were assigned an descriptor e.g. Doctor 2, to enable comparison of experiential perspectives to be made during analysis. In the findings these were changed to pseudonyms to protect confidentiality.

Researchers' Personal characteristics

During this study, Camilla (MPharmS) was a clinical mental health pharmacist in forensic services in the mental health trust where the study was conducted and the study formed part of her postgraduate MSc Research project. She completed relevant theoretical and experiential training in qualitative research methods, particularly in interpretative phenomenological analysis. CS completed all interviews and focus groups, and was observed on two occasions by Denise Taylor (DT) for appropriateness and effectiveness.

DT (PhD, MSc, MPharmS, FFRPS, MCMHP) was a senior lecturer and research supervisor for CS; providing training in qualitative research methods, analyses, informed consent and capacity assessment.

Relationship with participants

Due to her role as clinical pharmacist, CS had developed professional relationships with some (but not all) of the primary and secondary care HCPs prior to the study; she did not know any of the CSUs. All participants were aware this study was part of a further degree. IPA enables researcher expertise to be acknowledged through reflexivity, whilst simultaneously preserving the voice of the participant.

DAT knew none of the study participants but had previously worked in and researched delivery of clozapine services. Reflexivity throughout the study enabled personal and professional bias to be recognised and addressed.

Data analysis

For peer review only

As this research explored individuals' experiences and perceptions, interpretative phenomenological analysis (IPA) was used. The intention of IPA is to explore the participants' experience as perceived by them and reflect this in the interpretation by the researcher.[12] In line with accepted analytical process for IPA each transcript was manually coded line-by-line into themes by CS. Robustness of the analytical process was assessed by the coding of a transcript by both CS and DAT. There was a high level of agreement regarding coding and CS continued with the analytical process. At the end of the coding process CS and DAT met to discuss and clarify emerging themes. Once super-ordinate or 'master' themes were identified for each stakeholder participant group, the analysis moved to looking at connections and patterns across all participant groups. No software was used to aid the analysis of data or themes.

RESULTS

A total of thirty-eight participants were recruited; including 32 healthcare professionals (HCPs) and 6 CSU's (General Adult CMHT = 1; Forensic = 5), but no carers as no expressions of interest to participate were received. It is not known how many potential participants who were approached by members of the CMHT and decided not to participate in the study, but once recruited to the study there were no dropouts. 14 interviews and 6 homogenous focus groups from General Adult and Forensic CMHT's were completed. Demographics of participants can be found in Table 3.

Table 3: Demographics of Participants

Participants* per focus group or interview	Profession	Sector	Years with shared-care clozapine
Mark<br>Emily<br>Laura<br>Angela	Hospital Pharmacist<br>MMT[¥]<br>MMT[¥]<br>Receptionist	Hospital Pharmacy, Secondary care	1.5<br>2<br>5<br>5
Luke<br>Bethany<br>John	Community Psychiatric Nurses	Forensic Community Mental Health Team, secondary care	2<br>2<br>2
Kate<br>Anne<br>Denise	Community Psychiatric Nurses	General Adult Community Mental Health Team, secondary care	4<br>4<br>4
Jennifer<br>Trace<br>Victoria	Community Psychiatric Nurse Social Worker Community Psychiatric Nurses	General Adult Community Mental Health Team, secondary care	2<br>5<br>1.5
Lucy<br>Jane<br>Nick<br>Claire	All Social Workers	Forensic Community Mental Health Team, secondary care	2<br>2<br>2<br>2
Tim<br>Simon<br>Adam<br>Frank<br>Rob	All Clozapine Services Users (CSUs	Attached to the Forensic Community Mental Health Team (CMHT)	1-2<br>1-2<br>1-2<br>1-2<br>1-2

For peer review only

Richard	CSU	Attached to General Adult CMHT	unknown
Dr Harrison	Responsible Clinician	Forensic	2
Dr Brown	Responsible Clinician	Forensic	2
Dr Taylor	Responsible Clinician	Forensic	2
Dr Smith	Responsible Clinician	General Adult	4
Charles	Community pharmacist	Primary care	unknown
Catherine	Community pharmacist	Primary care	2-3
Margaret	Community pharmacist	Primary care	4
Tom	Community pharmacist	Primary care	9 months
Sophie	Community pharmacist	Primary care	unknown
Marie	Community pharmacist	Primary care	4-5
Dr Hudson	GP	Primary care	2
Dr White	GP	Primary care	10
Dr Green	GP	Primary care	2

* Pseudonym names used for confidentiality ¥ Medicines Management Technician

Duration of interviews and focus groups ranged from 30 to 90 minutes and none were repeated. Data saturation was achieved as no new themes emerged during the analysis of final transcripts and field notes were taken for each interview or focus group to support reflexivity. Due to limitations in time and funding, transcripts and findings were not returned to participants for comment. The findings below describe participant perceptions of their experiences of a shared-care clozapine service.

Four superordinate themes emerged from the data; The Sharing of Care, Multi-professional Relationships, The Provision of Care and the Clozapine Process. The inter-connectedness of these themes and minor themes is illustrated in Figure 1.

Excerpts from participant transcripts (shown in indented italic text) have been chosen to exemplify experiential perceptions and changed as little as possible to retain data authenticity. If additions were made for clarification these are bracketed within the quote and non-italicised. When the term Forensic HCP or General Adult HCP is used, Forensic and General Adult CMHT HCP is being referred to respectively.

1. Clozapine Process

For peer review only

Clozapine is a unique antipsychotic with regard to its prescribing, monitoring and supply; therefore knowledge of all the components of prescribing clozapine and its possible consequences, are important factors in a clozapine supply service.

1.1 Knowledge

The process involved in prescribing and supplying clozapine is different to any other antipsychotic, therefore it takes time and experience to understand and become familiar with its nuances. Greater participant knowledge of the process meant that HCPs understood why things might go wrong, enabling them to have greater confidence in resolving these issues and their role within that.

> *"I looked up some information and used the BNF and it was I guess somewhat hesitant, clearly it is medication that has got some significant potential side effects and risks with it. But umm I think that I felt like I have got a good working relationship with the mental health team and working with the patient that I have on clozapine, and so was able to talk with the* (hospital) *pharmacist and address any issues." Dr Green, GP*

> *"I suppose it's enlarged my experience so I'm more confident with ……once you do more than one customer your confidence increases and you find out the….and you can deal with customers easier can't you? So professionally that way and also you're you feel confident to deal with those patients and other professionals as well" Marie, Community Pharmacist*

1.2 In Practice

Initially HCPs perceived the CSU required certain skills such as organising and planning for shared care clozapine to be successful, so they used surrogate markers of stability and capability to identify suitability of CSUs. With experience of working with shared care, Forensic HCPs realised that CSUs didn't need to be already stable or capable in order for shared care to be successful. They found CSUs responded well to the challenge of being responsible for obtaining their own FBC, repeat prescription and supply of clozapine.

> "[A (CSU) *as well who was really chaotic initially, and we were really worried about whether he'd manage it all, but it's actually made him get a lot more organised." Bethany, Forensic CPN*

HCPs and CSUs perceived the timeframe between obtaining a valid FBC and supplying clozapine before the last supply ran out as being very tight. This became a source of anxiety for both HCPs and CSUs, as they were extremely aware of the potential negative consequences of readmission to hospital for re-titration with a delayed supply of clozapine. Circumstances which could delay the supply of clozapine were perceived to occur relatively frequently by community pharmacists.

> *"….I guess it's just because there's such a tight schedule of the drug, it gives you no leeway for these kind of exceptional circumstances which do happen reasonably regularly." Charles, Community Pharmacist*

Limitations to GP appointment systems were identified, as for some it was not possible to book an appointment 4 weeks in advance for a FBC. This meant CSUs needed to adapt their routine to ensure they remembered to book their appointment at a later date and when sufficient appointments were still available.

For peer review only - http://bmjopen.bmj.com/site/about/guidelines.xhtml

> *"They give it up to like…I think it's 2 weeks maximum but they won't do a full 28 days, but like* (I) *write down on a calendar today every month when my calendar starts, need to book the blood test and normally for the week beforehand to book it. " Adam, CSU*

2. The Sharing of Care

The sharing of care reverberated far wider than just the prescribing and monitoring of clozapine and was associated with its own obstacles as it required individuals to work in a different way.

2.1 Reverberations of shared care

Some HCPs thought that the drive for shared care clozapine was one of cost saving, however with experience, Forensic HCPs identified greater CSU independence as their driver for engagement. For Forensic HCPs shared care not only enabled CSUs to continue to develop skills required for independence, but also a new opportunity for them to demonstrate trust in the CSU. This trust led to a change in the relationship dynamic and activated the CSU to take responsibility for their care and engage in the process.

> *"Initially it was savings because….quite some time once I saw how the first person was working and did….once I saw the difference it made with him, how then we felt about him taking medication and how he was responding to that, I think then I moved away from seeing it as a cost saving exercise and seeing it as something that could actually useful in maintaining independence for the client." Nick, Forensic SW*

This was supported by CSUs views that shared-care was a supportive opportunity to develop their independence and take back ownership of their health, which they felt in turn boosted their self-esteem. CSUs also perceived that engaging in shared care was normalising and reduced stigma associated with mental illness and clozapine treatment.

> *"The emphasis is all on you sort of thin in the community, whereas before it was thought that people with mental illness couldn't live in the community properly without having problems and things like that, whereas people are seeing especially the people on Clozapine, that they can live a good normal active life in the community…" Adam, CSU*

The physical health of CSUs was valued as equally important to their mental health and shared care provided GP involvement in clozapine prescribing with awareness of the need to manage negative effects of clozapine on physical health. Both Forensic HCPs and CSUs perceived the GP as the expert in physical health and appreciated and benefitted from, this more holistic approach to care. In contrast, GPs not involved in shared care may not even be aware their patient receives clozapine from a mental health trust and any possible associated negative iatrogenic effects. At the time of this study, shared care records between primary and secondary care were not widely used.

> *"You can't separate off the, you know in the case of these patients, the mental health issues from the physical health and their overall wellbeing, emotional spiritual wellbeing, it is all their whole entity part of the whole and interplay with one another." Dr Green, GP*

2.2 Obstacles to working in a different way

For peer review only - http://bmjopen.bmj.com/site/about/guidelines.xhtml

Shared care clozapine required secondary care HCPs to trust primary care HCPs they may not have previously met, and for primary care HCPs to take on the responsibility of an unfamiliar medication with very restrictive prescribing and supply requirements. As mentioned in 1.2, there was initial apprehension on possible negative consequences of a missed clozapine dose. Apprehensions from primary care HCPs eased over time due to growing relationships with secondary care HCPs providing a sense of support and a true sharing of care.

> *"It's not a drug I'm totally at ease with, so there are apprehensions when any specialist says 'please do this, these are the protocols' and unless you really feel you've got a backup, you feel somewhat exposed. That hasn't particularly been an issue because the support has been there." Dr White, GP*

Often General Adult HCPs described negative assumptions on the abilities and/or expectations of CSUs in relation to independence and involvement in managing their own medication. This affected their ability to trust CSUs in taking on this responsibility or even seeing a need for shared care.

> *"I suppose* (it) *depends how well they are doesn't it, how insightful they are in the first place, how sassy they are with how systems work and stuff really. At the end of the day, they just want their meds to turn up when they need more. (The CPN) comes round at 4 with the meds, that's what they want isn't it?" Jane, General Adult CPN*

3. The Provision of Care

Although clozapine is the active treatment, the care provision included the role each member of the multi-professional team played in supporting the recovery and management of the individual.

3.1 Clozapine as a treatment

Many healthcare professionals and all CSUs thought that clozapine often provided the best opportunity for recovery and hospital discharge in individuals with schizophrenia; particularly if they had already tried other antipsychotics without success. These highly valued benefits of clozapine provided the motivation for CSUs to continue taking it despite its negatives effects.

> *"The other thing with Clozapine which was explained to me is side effects and pluses and minuses and sort of thing, but it's like to me it's takin' something that keeps me well. I class it the same as eating food and drinking water. I need that to keep me level and keep me… I feel totally normal now whereas I couldn't before and I was on all of the old medications* [….] *whole range of different and they never really worked. Clozapine was the first one that ever worked. The only downside to it was the side effects; the dribble, makes you sleep, makes you weight gain, things like that but when you weigh up the good and the bad Clozapine is up there it does the job." Adam, CSU*

3.2 Playing my part

Forensic HCPs appreciated the therapeutic value of supporting CSUs to be as independent as possible through normalisation and socialisation. They perceived that a large part of their role was to work with and support CSUs in this way, rather than focussing solely on the therapeutic use of medication.

For peer review only - http://bmjopen.bmj.com/site/about/guidelines.xhtml

> *"A large part of our job isn't just about giving medication to people.* (What) *I quite like about my job is about socialisation and normalisation and trying to get people to live in a normal way." John, Forensic CPN*

In contrast, General Adult HCPs perceived a sense of responsibility for co-ordinating the process of clozapine supply and undertook as much of the process as possible on behalf of the CSU. They only saw shared care clozapine as an alternative method of obtaining a supply of clozapine.

> *"Difficult I would say, very difficult, lots of chasing finding about results, seeing if the pharmacy understands you and knows what you're trying to ask for, and then trying to establish whether they've actually got the medication or not, when they're going to get it then you've got to faff around, pick it up and get it to the patient it's a nightmare." Kate, General Adult CPN*

GPs perceived their main role in shared-care to be prescribing clozapine, checking FBC results, and CSU wellbeing and functioning. However they also spent time liaising with primary and secondary care team members with respect to any CSU or clozapine-related problems.

> *"…so there's the practicalities of just the physical monitoring of whether the bloods are ok umm there is a responsibility just to see the patient is ongoing wellbeing and mental health is stable and they're functioning. The responsibility to support the people taking the blood and if they're concerned, they're obviously not qualified, don't have the expertise to make any sort of mental health type assessment, so being available for them to be able to come and say this isn't right worried about this and then obviously responsibility for liaising with the mental health team if there are concerns or worries." Dr White, GP*

In order to supply clozapine on prescription, community pharmacists require all previous steps of the process to be completed. In practice, community pharmacists often found they were heavily involved in identifying and co-ordinating missing elements of the process which prevented them completing their dispensing role.

> *"So we do all the chasing about to find out what stage we're at with all these things because if you phone Denzapine they'll say 'oh we haven't had a result in yet' and then you have to phone the surgery say, 'has this person had a blood test' or get in touch with CPN so we're doing a lot of chasing around." Charles, Community Pharmacist*

CSUs perceived their role in shared-care was to obtain a FBC from the GP surgery, order their prescription, and collect and take, their clozapine. Many CSUs used services provided by community pharmacy such as repeat prescriptions and delivery to support them in undertaking their role in shared care.

> *"Yeah it all good for me now on, so all I've got to do is every 28 days have my bloods done and then they* (community pharmacy) *do the rest and that, so I don't have to sign no paper or run around no more I've just got to go to the GP surgery and see the nurse and have blood samples." Simon, CSU*

4. Multi-professional Relationships

For peer review only - http://bmjopen.bmj.com/site/about/guidelines.xhtml

Being recognised as a member of a particular team supported the development of multi-professional relationships and communication. These relationships were integral to building trust and understanding of each other's role and the provision of peer support.

4.1 Recognition as a team member

Knowing the members of the team involved in the provision of shared care clozapine positively influenced communication and supported building relationships with both CSUs and HCPs being more comfortable to make contact with someone if they knew them. This increasing familiarity led to increased involvement and contact which developed relationships still further.

> *"I think if there was a problem with one of my guys in the* (community) *pharmacy where I've been in I'll actually go in and talk to him* (community pharmacist) *now whereas before I'd come through* (the hospital pharmacy) *so I talk to him now*." *John, Forensic CPN*

Through building a relationship individuals were increasingly able to understand the expertise and role each team member held, which supported identifying the most appropriate person to contact for support.

> *"I mostly do things to do with my mental health with my consultant and things to do with my physical health with my GP." Richard, CSU*

4.2 Supporting each other

Relationships were a key element in feeling supported and building trust. Support ranged from provision of information or advice to provision of additional resource when needed. The sense of feeling supported was characterised through a reliable relationship which was responsive to need.

> *"Knowing that there is somewhere that you can call on is important and certainly the particular community mental health nurse who is involved in the care she is excellent. Apart from these meetings she is available for discussion and she has come along and organised blood tests and like if there seems to be a more urgent situation where some compliance has gone wrong."*
> *Dr White, GP*

Sharing the responsibility of clozapine provision through shared-care was seen as a source of support for primary care by GPs and RCs, as it reinforced a sense of an equally shared responsibility and supported the ability to work in a multi-disciplinary way.

> *"Well it enables us to umm to manage this difficult client group for patients that need to take it, so we are happy to accept our share of the responsibility for that and…. so it is a positive thing and it helps us with our sort of multi-disciplinary work."* *Dr Hudson, GP*

DISCUSSION AND CONCLUSIONS

Principle findings

All participants perceived clozapine provision as different to other antipsychotics because of the process of supply, its beneficial and negative effects, greater reliance on members of the multi-

For peer review only

professional team and system processes. Greater knowledge and understanding of clozapine provision was generally obtained through experience and led to individuals having more confidence in their own role and resolving issues that arose. Developing multi-professional relationships was key in building trust, understanding roles, improving communication and feeling supported. Shared care reinforced a sense of equal and shared responsibility between primary and secondary care; this supported further development of multi-professional relationships and was seen as a source of support. Addition of a GP to the team, provided more holistic care for the CSU enabling physical health interventions and improved GP awareness that their patients were taking clozapine. This awareness decreased potential iatrogenic health risks associated with clozapine.[13]

Surrogate markers of stability and capability were originally used to identify CSUs who were potentially suitable for clozapine shared care. However with experience Forensic HCPs identified shared care as a new opportunity to demonstrate trust in the CSU and support them to develop skills of self-dependence and independence through normalisation and socialisation. This reduced Forensic HCPs reliance on surrogate markers. Engaging in shared care clozapine led to a change in the relationship dynamic between the Forensic HCPs and CSUs, which in turn enabled the CSU to take responsibility and ownership for their care. In contrast, General Adult HCPs held paternalistic assumptions of CSU abilities and/or expectations in relation to their independence and involvement in managing their own medication. This affected General Adult HCPs ability to trust CSUs in possibly taking on this responsibility or perceiving a need for shared care clozapine.

Implications for Practice

Relationships in healthcare provision

In 2010, the Health Foundation launched 'Closing the Gap through Changing Relationships', a programme which focused on recognising the need to change the way healthcare systems work by challenging beliefs and behaviours of healthcare workers to improve quality of care. Evidence demonstrates that best health outcomes and experiences are achieved when people have an active part in their own care and receive responsive support according to their needs. Being able to play an active role is a consequence of the dynamic created by the manner in which support is provided.[14] The programme also explored relationships between service users and providers in healthcare settings using a conceptual framework proposed by Wish et al,[14,15] which identified four primary relationship dimensions to describe the character of different relationships. These are power, valence, intensity and formality.[15]

We mapped the HCP-CSU relationships in Forensics and General Adult to the above relationship dimensions to explore how shared care clozapine influenced these HCP/CSU relationships. See Table 4 for further detail.

For peer review only

Table 4: Mapping Relationships to the Wish Model

The Wish Model Relationship Continuum Components		The Relationship Dynamic	
		**Forensic CMHT HCPs**	**General Adult CMHT HCPs**
Asymmetric --- Symmetric	**Power**<br>dependence<br>centredness<br>agency	Greater sense of partnership for both CSUs and HCP.<br><br>Themes centred around the CSU rather than the HCP.<br><br>CSUs have gained greater agency and reduced dependence on HCPs.<br><br>**Result: perception of a symmetrical HCP/CSU relationship.**	HCPs complete activities either for or on-behalf of CSU.<br><br>Themes centred on HCPs rather than CSU.<br><br>CSU appears to be highly dependent on the HCP.<br><br>**Result: perception of an asymmetrical HCP/CSU relationship.**
Competitive --- Cooperative	**Valence**<br>agreement<br>communication<br>conflict	HCP & CSU agendas aligned to increase independence and normalisation.<br><br>Engagement in shared care provided an opportunity to achieve their shared agendas.<br><br>HCPs trust in CSUs ability to perform own role in shared care.<br><br>**Result: perception of a co-operative HCP/CSU relationship.**	No evidence of agenda alignment.<br><br>Formal medical knowledge competing with lay knowledge.<br><br>Some HCPs described lacking trust in CSU relationships.<br><br>**Result: perception of a competitive component to HCP/CSU relationship.**
Distant --- Intimate	**Intensity**<br>transactional/ relational<br>commitment	Monitoring prescription collection transactional element but in context of a relational focus on a CSU long-term goals.<br><br>Forensic HCPs demonstrated commitment through motivation and willingness to adapt their beliefs and behaviours to fulfil the shared agenda of independence. CSUs committed to their own role in shared care.<br><br>**Result: perception of an intimate HCP/CSU relationship.**	Adult HCPs talked about undertaking activities on behalf of the CSU with no reference to the future aims of the CSU.<br><br>Adult HCPs demonstrated little change in beliefs and behaviours in a shared care role of the CSU's; suggesting a distant relationship with little commitment for the potentially longer term goals of the CSU.<br><br>**Result: perception of a transactional and distant HCP/CSU relationship.**
Professional --- Social	**Formality**<br>affect<br>exclusivity	Engaging in shared care enabled a shift in formality dimension to social as the relationship extends into primary care.<br><br>Social affect aided by the commitment to HCP-CSU relationship.<br><br>**Result: perception of a more social professional HCP/CSU relationship.**	Considerable professional formality.<br><br>Professional affect demonstrated by the power differential between the HCP and CSU.<br><br>**Result: perception of a formal professional HCP/CSU relationship.**

For peer review only

General Adult HCPs appeared to hold negative assumptions of the abilities and/or expectations of CSUs managing their own medication. These assumptions reinforced their inherent paternalistic role demonstrated by taking responsibility for the CSU obtaining clozapine, which created dependence. Shared care clozapine appears to have had no influence in supporting changes in the General Adult HCP-CSU relationship dimensions and so it continues to be characterised by asymmetric, competitive, distant and professional qualities. This may provide an explanation as to why General Adult HCPs did not appear to see a need for shared care clozapine or invite CSUs to participate in this study.

In contrast, the effect of shared care clozapine on Forensic HCP-CSU relationship, influenced change in all four Wish dimensions. Engaging in shared care clozapine enabled a shift in the power differential between HCP and CSU to a more symmetric and co-operative approach, possibly through the ability to trust in each other and share a common agenda of CSU independence. The willingness to engage in new ways of working, enabled them to tailor their support for CSUs to continue to develop skills for independence, demonstrating commitment to the long term agenda of independence and relationship integrity. Shared care clozapine appeared to cultivate changes in the Forensic HCP-CSU relationship dimensions which enabled CSUs to expand their ability to live independent and fulfilling lives; key components of person-centred care.[16] In contrast the General Adult HCP-CSU relationship is reflective of a biomedical l model and they described a more negative experience of shared care compared to the more positive experience of Forensic HCPs.[17]

Person-centred care

Providing care which is person-centred is dependent on ensuring care affords people dignity, respect and compassion; care, support or treatment which is coordinated, personalised and enables patients to live an independent and fulfilling life.[16]

Key principles of person-centred care with particular relevance to successful shared care clozapine outcomes include knowing the patient as a person, recognising their individuality and expertise in their own health and care and taking a holistic approach to assessment and provision of care which is centred around them. Importantly staff need to be supportive, share the power and responsibility of care and be well trained in communication.[18]

Engagement in shared care clozapine with a relationship demonstrated by the Forensic HCP/CSU partnership, provides an example of a shift in care from paternalistic to person-centred. We suggest that shared care clozapine in this context, offers an opportunity to demonstrate dignity, respect and compassion to CSUs through trust and handing over greater responsibility to the CSU. The provision of coordinated care and support through the development of relationships between multi-professional team members in primary and secondary care enabled personalised care and support for CSUs, which was subsequently reflected in CSUs developing their ability to manage their own role in shared care. This person-centred approach ultimately enabled CSUs to live an independent and fulfilling life by self-managing their own clozapine.

Dartmouth Atlas defines effective care as *"services that are of proven value and have no significant tradeoffs"*[19] Based on CSUs and Forensic HCPs experiences, shared care clozapine is effective for these participants. Currently there is no definition of acceptability in healthcare, although Sekhon et al (2017), propose a theoretical yet to be validated framwork.[20] We suggest that acceptability of shared care clozapine is demonstrated by the active engagement of CSUs and Forensic HCPs in the process.

For peer review only - http://bmjopen.bmj.com/site/about/guidelines.xhtml

Future research

Future research using implementation theory is needed to understand in greater detail, the barriers and enablers to wider implementation of shared care clozapine to support integration of people with serious mental illness into the community.[21,22]

Strengths and limitations of this study

This is the first qualitative study to explore individual perceptions on experiences of shared-care clozapine service by those that use and provide it. Identification of potential participants relied on two information sources being up-to-date; the Denzapine® Monitoring Service website and electronic medical notes. CSU participants were not equally represented from Adult and Forensic CMHTs, which may mean experiences and perceptions of General Adult CSUs are not sufficiently represented. Recruitment of CSUs was dependent on invitation for participation from their own clinical team. Greater recruitment of Forensic CSUs could relate to their enabling approach to care or a reflection of the team's experience of shared-care in comparison to General Adult CMHT. No carers of CSUs participated, possibly reflecting the isolation that some people with serious mental illness live with.

Interpretative Phenomenological Analysis methodology guided the development of topic guides, completion of interviews and homogenous focus groups used to collect the qualitative data. IPA also allowed the experience of the researchers to be noted in the reflexivity associated with data analysis. Quality assurance processes were completed to provide validity to this research, for example piloting topic guides, supervised focus groups and joint co-coding and re-coding of data. Data validity is reflected in the ability to substantiate the analysis with participant quotes and provide a dialogue of discussion between the results and existing literature.

In conclusion, mental health has seen an increase in demand on limited health service resources with cuts in funding. This has led to a number of trusts embarking on transformative programmes in order to reduce costs, shift demand from acute services, and deliver care focused on recovery and self-management.[23] Person-centred care is recognised nationally as one way in which this can be achieved through a substantial effect on quality of care.[16,18] Our results suggest shared care clozapine could be one way of expanding person-centred care in mental health which the literature demonstrates demonstrable improvements in quality of care, integration into the community and reductions in stigma and healthcare resources.

Figure 1: Illustration of superordinate themes and their connections

Acknowledgements

This study was funded by a small grant from the College of Mental Health Pharmacy. We thank all study participants for their input into this study and for the team members who helped in identification of potential participants.

For peer review only - http://bmjopen.bmj.com/site/about/guidelines.xhtml

REFERENCES

1 DH. *Equity and excellence: Liberating the NHS*. July 2010.
2 HMG/DH. *No Health Without Mental Health: A Cross-Government Mental Health Outcomes Strategy for People of All Ages*. February 2011.
3 NHS England. *Five Year Forward View*. October 2014.
4 Cowen P, Harrison P, Burns T. *Shorter Oxford Textbook of Psychiatry* (Chapter 11, Schizophrenia). Oxford University Press, 2012: 255-296.
5 Kane J, Honigfeld G, Singer J, Meltzer H, The Clozaril Collaborative Study Group. Clozapine for the treatment-resistant schizophrenic: results of a US multicentre trial. *Psychopharmacology*. 1989; 99: S60-S63.
6 Britannia Pharmaceuticals. *Denzapine 100mg tablets - Summary of Product Characteristics* (15th January 2015). http://www.medicines.org.uk/emc/medicine/25965 (accessed 11 Jun 2017).
7 Filia S, Lee S, Sinclair K, Wheelhouse A, de Castella A et al. Demonstrating the effectiveness of less restrictive care pathways for the management of patients treated with clozapine. *Australasian Psychiatry*. 2013; 21(5): 449-455.
8 Filia S, Wheelhouse A, Lee S, Main M, de Castella A, Wilkins S et al. Transitioning patients taking clozapine from the public to private/GP shared-care setting: Barriers and criteria. *Australian and New Zealand Journal of Psychiatry*. 2012; 46(3): 225-231.
9 Kelly B, Perkins D, Fuller JD, Parker S. Shared care in mental illness: A rapid review to inform implementation. *International Journal of Mental Health Systems*. 2011; 5: 31.
10 Smith SM, Allwright S, O'Dowd T. Effectiveness of shared care across the interface between primary and speciality are in chronic disease management. *Cochrane Database of Systematic Reviews*. 2007.
11 Smith JA, Osborn M. Chapter 4 *Interpretative Phenomenological* Analysis in Qualitative Psychology. A Practical Guide to Research Methods. Ed Smith JA. Sage Publications, London 2003.
12 Smith J, Flowers P, Larkin M. *Interpretative phenomenological analysis: Theory, Method and Research*. Sage, 2013.
13 Murphy K, Coombes I, Moudgil V, Patterson S, Wheeler A. Clozapine and concomitant medications: Assessing the completeness and accuracy of medication records for people prescribed clozapine under shared care arrangements. *J Eval Clin Pract.* Published Online First: 4 May 2017. Doi: 10.1111/jep.12743
14 The Health Foundation. *Closing the gap through changing relationships: evaluation*. June 2014. London, The Health Foundation. http://www.health.org.uk/sites/health/files/ClosingTheGapThroughChangingRelationshipsEvaluation.pdf (last accessed20-06-2017)
15 Wish M. Comparisons among multidimensional structures of interpersonal relations. *Multivariate Behav Res*. 1976; 11(3): 297-324.
16 The Health Foundation. *Person-centred care made simple*. 2014. London. The Health Foundation. http://www.health.org.uk/sites/health/files/PersonCentredCareMadeSimple.pdf (Last accessed 20-06-2017)
17 Engel G. The Need for a New Medical Model: A Challenge for Biomedicine. *Science*. 1977; 196: 129-196.
18 Health Innovation Network: South London. What is person-centred care and why is it important?. http://www.hin-southlondon.org/system/ckeditor_assets/attachments/41/what_is_person-centred_care_and_why_is_it_important.pdf (accessed 11 Jun 2017).
19 Anon. Effective Care. A Dartmouth Atlas Project Topic Brief. May 2007. Centre for Evaluative Clinical Services. http://www.dartmouthatlas.org/downloads/reports/effective_care.pdf (accessed 20 06 2017).
20 Sekhon M, Cartright M, Francis J. Acceptability of healthcare interventions: an overview of reviews and development of a theoretical framework. *BMC Health Services Research.* Published Online First: 26 January 2017. doi: 10.1186/s12913-017-2031-8
21 May C, Finch T. Implementing, Embedding and Integrating Practices: An Outline of Normalization Process Theory. *Sociology*. 2009; 43(3): 535-554.
22 Greenhalgh T, Robert G, MacFarlane F et al. Diffusion of innovations in service organisations: Systematic review and recommendations. *The Milbank Quarterly*. 2004; 82(4): 581-629.

For peer review only

23 The Kings Fund. Briefing: Mental health under pressure. https://www.kingsfund.org.uk/sites/files/kf/field/field_publication_file/mental-health-under-pressure-nov15_0.pdf (accessed 11 Jun 2017).

For peer review only

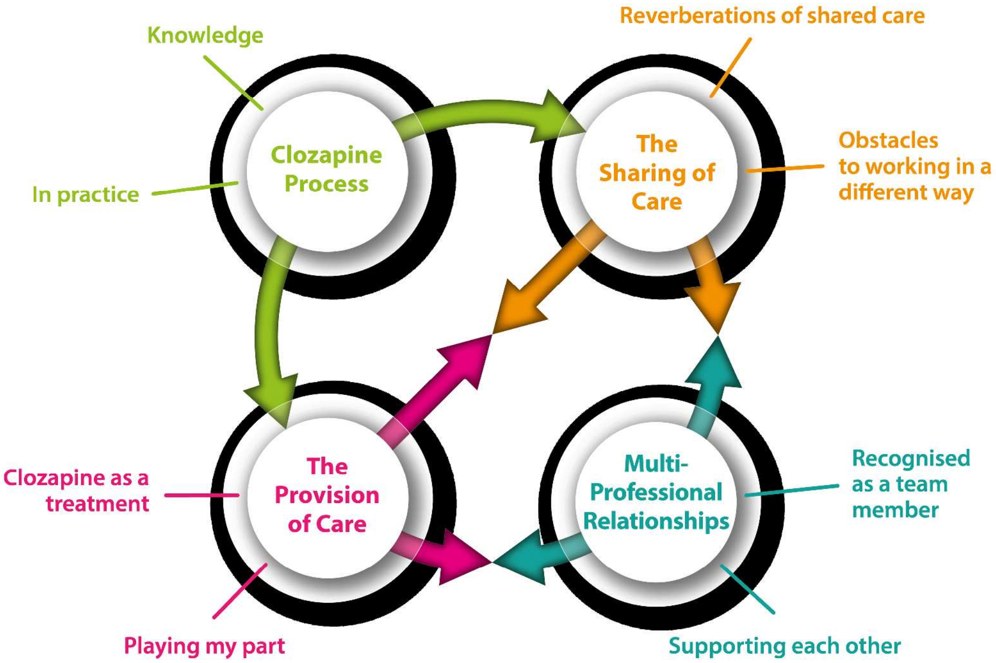

For peer review only

COREQ:

Domain One: Research team and reflexivity

**No.**	**Item**	**Guide questions/description**	**In Paper**
**Personal Characteristics**			
**1**	Interviewer/Facilitator	Which author conducted IV/FGs?	Yes: p6 lines 15-17
**2**	Credentials	What were the researcher's credentials? *E.g. PhD, MD*	Yes: p6 lines 25-34
**3**	Occupation	What was their occupation at the time of the study	Yes: p6 lines 25-34
**4**	Gender	Was the researcher male or female?	Yes: p6 lines 25-34
**5**	Experience & training	What experience or training did the researcher have?	Yes: p6 lines 25-34
Relationship with participants			
**6**	Relationship established	Was a relationship established prior to study commencement?	Yes: p6 lines 35-43
**7**	Participant knowledge of the interviewer	What did the participants know about the researcher? e.g. *personal goals, reasons for doing the research*	Yes:p6 lines 35-43
**8**	Interviewer characteristics	What characteristics were reported about the interviewer/facilitator? e.g. *Bias, assumptions, reasons and interests in the research topic*	Yes: p6 lines 35-43

Domain Two: Study Design

**No.**	**Item**	**Guide questions/description**	**In Paper**
Theoretical framework			
**9**	Methodological orientation and Theory	What methodological orientation was stated to underpin	Yes: p4 lines 28-31

For peer review only

		the study? *e.g. grounded theory, discourse analysis, ethnography, phenomenology, content analysis*	
Participant selection			
**10**	Sampling	How were participants selected? *e.g. purposive, convenience, consecutive, snowball*	Yes: p6 lines 3-6
**11**	Method of approach	How were participants approached? e.*g. face-to-face, telephone, mail, email*	Yes: p6 lines 7-14
**12**	Sample size	How many participants were in the study?	Yes: p7 lines 13-15
**13**	Non-participation	How many people refused to participate or dropped out? Reasons?	Yes: p7 lines 15-17
**Setting**			
**14**	Setting of data collection	Where was the data collected? e.*g. home, clinic, workplace*	Yes: p6 lines 15-17
**15**	Presence of non-participants	Was anyone else present besides the participants and researchers?	Yes: p6 lines 17-19
**16**	Description of sample	What are the important characteristics of the sample? *e.g. demographic data, date*	Yes: p7 line 18 and Table 3
**Data Collection**			
**17**	Interview guide	Were questions, prompts, guides provided by the	Yes: p5 Table 2 lines 1- 44,<br>P6 line 1-2

Comment [SC1]: Not sure where we have put anything?

Comment [DT2]: Reply see green sentence P7 line 15-16

For peer review only

		authors? Was it pilot tested?	
**18**	Repeat interviews	Were repeat interviews carried out? If yes, how many?	Yes: p8 line 2
**19**	Audio/visual recording	Did the research use audio or visual recording to collect the data?	Yes: p6 lines 20-21
**20**	Field notes	Were field notes made during and/or after the interview or focus group?	Yes: p8 line 4
**21**	Duration	What was the duration of the interviews or focus group?	Yes: p8 lines 2-3
**22**	Data saturation	Was data saturation discussed?	Yes: p8 line 2
**23**	Transcripts returned	Were transcripts returned to participants for comment and/or correction?	Yes: p8 lines 4-5

Domain 3: Analysis and Findings

**No.**	**Item**	**Guide questions/description**	**In Paper**
**Data analysis**			
**24**	Number of data coders	How many data coders coded the data?	Yes: p7 lines 4-7
**25**	Description of the coding tree	Did authors provide a description of the coding tree?	Yes: p8 lines 8-10 and Figure 1
**26**	Derivation of themes	Were themes identified in advance or derived from the data?	Yes: p8 lines 5-7
**27**	**S**oftware	What software, if applicable, was used to manage the data?	Yes: p7 lines 9-10
**28**	Participant checking	Did participants	Yes: p8 lines 4-5

Comment [SC3]: We don't actually mention that I did all the coding….agree this needs to be added?

Comment [DT4]: I added your initials to a sentence and changed the order to make it clearer

For peer review only

		provide feedback on the findings?	
**Participant Checking**			
**29**	Were participant quotations presented to illustrate the themes / findings?	Were participant quotations presented to illustrate the themes / findings? Was each quotation identified? e.g. *participant number*	Yes: p8 lines 11-13 and throughout results section, example p9 lines 9-14
**30**	Data and findings consistent	Was there consistency between the data presented and the findings?	Yes: p8 lines 11-12, and throughout results section
**31**	Clarity of major themes	Were major themes clearly presented in the findings?	Yes: p8 lines 8-10
**32**	Clarity of minor themes	Is there a description of diverse cases or discussion of minor themes?	Yes: p8 line 10 demonstrated in Figure 1

For peer review only

BMJ Open

Cross Sector User and Provider Perceptions on Experiences of Shared Care Clozapine – a qualitative study

Journal:	*BMJ Open*
Manuscript ID	bmjopen-2017-017183.R2
Article Type:	Research
Date Submitted by the Author:	03-Aug-2017
Complete List of Authors:	Sowerby, Camilla; Universtiy of Bath, Pharmacy and Pharmacology<br>Taylor, Denise; Universtiy of Bath, Pharmacy and Pharmacology
**Primary Subject Heading**:	Mental health
Secondary Subject Heading:	Qualitative research, Patient-centred medicine, Health services research, General practice / Family practice
Keywords:	MENTAL HEALTH, QUALITATIVE RESEARCH, Schizophrenia & psychotic disorders < PSYCHIATRY

SCHOLARONE™
Manuscripts

TITLE PAGE

Title: Cross Sector User and Provider Perceptions on Experiences of Shared Care Clozapine – a qualitative study

Mrs Camilla Sowerby and Dr Denise Taylor

Corresponding author: Mrs Camilla Sowerby, Early Intervention in Psychosis Pharmacist, The Old School House School Lane Aston Rowant, OX49 5SU, Camilla.sowerby@berkshire.nhs.uk, 07876216828

Co-author: Dr Denise Taylor, Senior Lecturer, Department of Pharmacy and Pharmacology, University of Bath, Bath, UK. D.A.Taylor@bath.ac.uk

Key words: Shared-care, Clozapine service, Primary care, Cross sector working, Multi-professional working

Word count 5783 words

Author Contribution

Camilla Sowerby and Denise Taylor

- Substantial contributions to the conception, design; and acquisition, analysis, and interpretation of data for the work; AND
- Drafting and critically revising the work; AND
- Final approval of the version to be published; AND
- Agreement to be accountable for all aspects of the work in ensuring that questions related to the accuracy or integrity of any part of the work are appropriately investigated and resolved.

Competing Interests

Both authors have completed the ICMJE uniform disclosure form at www.icmje.org/coi_disclosure.pdf and declare: Mrs. Sowerby reports grants from College of Mental Health Pharmacy (CMHP), during the conduct of the study. Dr. Taylor reports that at the time of the grant being awarded was a council member of the CMHP, but the award allocation was judged by a grant advisory panel with due process and also completed two advisory boards for Sunovion (Lurasidone) during this time; and is a trustee for Bath Mind.

Funding

This work was supported by a small research grant awarded by the College of Mental Health Pharmacy.

Data sharing

All data collected during this study has been included in the analysis and is available for secondary analysis on request.

For peer review only

ABSTRACT (n= 304)

Objectives: 1) To explore individual perceptions on experiences of people receiving and/or delivering a shared-care clozapine service. 2) To gain an understanding of effectiveness and acceptability of shared-care clozapine.

Design: Interpretative phenomenological analysis guided the delivery and analysis of a semi-structured interview and focus group study designed to explore participant experience of shared care clozapine. Ethical approval 13/EM/0286 was gained in July 2013 from East Midlands – Nottingham 1 REC.

Participants: Eight stakeholder groups from Adult and Forensic Mental Health involved in shared care clozapine provision delivered in primary care, were identified for recruitment from one mental health trust in England (six different groups of healthcare professionals (HCPs), clozapine service users (CSUs) and their carers. To be eligible for recruitment all potential participants had to be either providing, receiving or the carer of a person receiving clozapine by shared care.

Results: 32 HCPs and 6 CSUs were recruited and 14 interviews and 6 participant homogenous focus groups were run. Four shared superordinate themes were identified; Clozapine Process, The Sharing of Care, The Provision of Care and Multi-professional Relationships. Differences between Adult and Forensic engagement in shared care were noted and both HCP-CSU relationship were mapped to the Wish conceptual framework of relationships to provide insight into how shared care clozapine can provide a mechanism for provision of person-centred care, which was present in the Forensic HCP-CSU but not General Adult HCP-CSU relationship.

Conclusions: The Forensic HCP/CSU relationship demonstrated how cross-sector working through shared care clozapine can provide a mechanism for provision of person-centred care by enabling a person-centred focus to care delivery which supported CSUs to live as independently as possible. Person-centred care demonstrably improves patient care outcomes and wider implementation of shared care clozapine could provide greater integration of people with serious mental illness and reduce stigma within the community whilst improving patient outcomes.

Strengths and limitations of this study:

- This is the first qualitative study to explore the experiences and perceptions of those that use and provide a shared-care clozapine service.
- Identification of potential participants relied on two information sources being up-to-date; the Denzapine® Monitoring Service website and the electronic medical notes.
- In terms of group size, CSUs were not equally represented in number, there were more Forensic CMHT CSUs compared to General Adult CMHT CSUs. This may mean the experiences and perceptions of CSUs from General Adult CMHT may not be sufficiently represented. The CSU recruitment method required them to be invited to the research by their own clinical team. The greater recruitment of CSUs from the Forensic CMHT could be related to their enabling approach to care or a reflection of the team's experience of shared-care in comparison to General Adult CMHT.
- No carers of CSUs participated, reflecting the isolation from family and loved ones that some people with serious mental illness live with.
- Data validity is reflected in the ability to substantiate the analysis with participant quotes and provide a dialogue of discussion between the results and existing literature.

For peer review only

INTRODUCTION (n=5578 – not including tables)

In recent years a number of United Kingdom (UK) government documents have been published concerning the provision of healthcare and mental health services, including: Equity and excellence: liberating the NHS,[1] No Health without Mental Health[2] and the Five Year Forward View.[3] The main focus of these publications is there should be choice in how patients obtain their care, and care should be individualised and recovery focused to improve patients' independence.

Clozapine is the only antipsychotic with efficacy in treatment resistant schizophrenia (TRS) and is effective in around one-third of these patients.[Error! Bookmark not defined.4,5] Additionally in the United Kingdom, due to the risk of clozapine-associated agranulocytosis, there is a requirement to undertake regular full blood count monitoring (FBC) and associated limitations on amount of medication supplied.[6] Additionally in the UK all prescribers, organisations dispensing clozapine and people taking clozapine, have to be registered with a brand specific clozapine website dependent on trust contract. An alternative model of clozapine provision to the usual clozapine clinic model or community mental health team (CMHT) based supply is shared-care clozapine (see Table 1).

Table 1: Description of models used to provide clozapine

	Community Mental Health Team (CMHT) based supply	Clozapine clinic	Shared care
FBC monitoring	The FBC monitoring is undertaken by GPs and either sent to the local pathology laboratory or posted to the clozapine company for processing. Results are automatically uploaded onto the DMS website if sent to them for processing, whilst hospital pharmacy upload those results of FBC's sent to the local pathology laboratory.	Clozapine patients attend the clinic, which is held at a hospital site, where FBC monitoring is undertaken and sent to the clozapine company for processing.	The FBC monitoring is undertaken by GPs and either sent to the local pathology laboratory or posted to the clozapine company for processing.
Prescription	The prescriptions are managed by hospital pharmacy and written by the Responsible Clinician of the clozapine patient. The patient maintains regular out-patient appointments with their RC. The frequency of these depend on the individual patient.	Same as CMHT-based supply.	The GP prescribed clozapine on an FP10 prescription. The CSU maintains regular out-patient appointments with their RC. The frequency of these depend on the Clozapine Service User (CSU). CSUs can only use the shared care service if they have been on clozapine for 12 months and are stabilised on treatment.
Dispensing	Hospital pharmacy dispenses clozapine to the relevant CMHT site or posts the medicine to the patient. Patients either collect their prescription from the CMHT site or CMHT staff to deliver to them.	Hospital pharmacy dispenses clozapine to the clozapine clinic. Patients collect their clozapine from the clinic.	Clozapine is supplied against the FP10 by the community pharmacy the CSU wishes to use. The community pharmacy needs to be registered with the clozapine company in order to be able to supply clozapine. CSUs either collect

For peer review only

			their prescription from the community pharmacy or utilise their delivery services.

METHODOLOGY AND METHODS

In this model clozapine service users (CSUs) obtain their FBC monitoring, prescription and clozapine supply from their GP and community pharmacy respectively. In shared-care the responsibilities for clozapine provision are shared between specialist mental health and primary care healthcare professionals. Evidence suggests that shared-care clozapine is effective in maintaining the monitoring and management of clozapine in a less restrictive care pathway.[7] Studies have highlighted that shared-care clozapine is not suitable for all CSUs and propose criteria to identify those most suitable.[7,8]

A growing body of evidence of the benefits of a shared-care approach in other long-term conditions including serious mental illness, is evolving.[9,10] Currently no published literature explores the perceptions of experiences of those that use and/or provide a shared-care clozapine service. The importance of patient experience of healthcare services is increasingly recognised in healthcare policy, to shape and inform service development, and payment structures for providers. Through understanding individuals' perceptions and experience of shared-care clozapine, insight can be gained into whether shared-care clozapine fulfils the government's vision for mental health care, and inform future service developments to improve quality and patient experience.

Aims of the study

1. To explore individual perceptions on experiences of people receiving and/or delivering a shared-care clozapine service.
2. To gain an understanding of effectiveness and acceptability of shared-care clozapine.

Ethical approval

This study took place within one National Health Service (NHS) mental health trust in the UK and a favourable NHS ethics committee opinion (East Midlands – Nottingham 1 NRES: Reference 13/EM/0286), and local Research and Development approval was received in 2013.

Study Design

Interpretative Phenomenological Analyses (IPA) was selected as the method as it attempts to explore the personal experience as it is perceived by the participant, rather than the researcher producing an objective statement of the event itself. IPA also allows for the expertise of the researcher to be acknowledged by reflexivity in the analytical processes.[11] Semi-structured interviews and focus groups methods were used to explore perceptions and experiences of clozapine service users (CSUs), their carers, general practitioners (GPs); community psychiatric nurses (CPNs), social workers (SWs), community and hospital pharmacy staff and responsible clinicians (RCs) (See Table 2 for topic guide).

For peer review only

Table 2: Focus group and interview topic guides

Clozapine Service User Topic Guide		Healthcare Professional Topic Guide	
Questions	Probing questions	Questions	Probing questions
When you were asked about participating in the shared care clozapine service, what were your first thoughts?	Why did you agree to participate in the clozapine shared care service?<br>How did it make you feel?<br>What does the clozapine shared care service mean to you?	What do you think the differences are in care for someone if they receive their clozapine through the shared care clozapine service compared to other means e.g. secondary care?	What do you think is the benefits and negatives are?<br>What do you think was trying to be achieved with the shared care agreement?<br>What do you think is actually achieved with the shared care service?<br>What does this mean for you?
What is your experience of being involved in the shared care clozapine service?	Describe the differences to you by obtaining your clozapine through the shared care service.<br>Can you identify positive and negative influences on your experience?<br>How does that make you feel?<br>What does that mean to you?<br>How has the shared care clozapine service impacted you?<br>How have your thoughts and feelings towards the clozapine shared care service changed over time?	What is your experience of being involved in the shared care clozapine service?	What were your first thoughts and feelings when you were asked to participate?<br>Can you identify positive and negative influences on your experience?<br>How does that make you feel?<br>What does that mean to you?<br>How has the shared care clozapine service impacted on you?
Describe the roles of the healthcare professionals that provide the clozapine shared care service.	How has the clozapine shared care service affected your thoughts and feelings about the healthcare professionals providing the clozapine shared care service?<br>Has the shared care service changed your relationship with these professionals?<br>In what way has the clozapine shared care service affected thoughts and feelings about these healthcare professionals?	Can you describe your role within the shared care clozapine service?	What does your input mean to you? How does it make you feel?<br>How has the shared care clozapine service affected your working relationship with other professionals?<br>What does this mean for you?<br>How has the shared care clozapine service developed you as a professional?<br>What training/support have you received? How did you receive this training?<br>What other ways would you like to get more involved in the care of these patients?

For peer review only

For peer review only - http://bmjopen.bmj.com/site/about/guidelines.xhtml

The HCP topic guide was piloted with pharmacist focus group participants and amended following feedback. Topic guides were also reviewed after each interview or focus group if questions were found to be too direct, leading or closed. To be eligible for recruitment all potential participants had to be either providing, receiving or the carer of a person receiving, clozapine by shared care. Carers of CSUs were only eligible for recruitment if the CSU they cared for invited them to participate in the research.

Eligible CSUs, RCs and community pharmacists were identified via the Denzapine® Monitoring Service (DMS) website. GPs were identified from CSUs electronic notes by the researcher (CS) and hospital pharmacist participants were identified by the dispensary manager. All General Adult and Forensic community mental health teams (CMHT's), were targeted by the researcher to potentially recruit CPNs and SWs working in shared-care services, and potential CSU participants were approached by their care team. All potential participants received an information pack about the study, including an expression of interest form and given a minimum of two weeks to make their decision.

All interviews and focus groups were to be completed by Camilla Sowerby (CS) and held at a convenient time and place for participants, either in a room at their place of work or on a mental health trust site, where participants could speak confidentially. Only researchers and participants were to be present during data collection and participant consent was to be received immediately prior to them taking part. Each participant received a gift voucher as a token of appreciation for their time and input into the study. Interviews and focus groups were audio recorded and transcribed verbatim to ensure a faithful reproduction of the participants' participation and anonymised by removing identifiers. Transcripts were assigned an descriptor e.g. Doctor 2, to enable comparison of experiential perspectives to be made during analysis. In the findings these were changed to pseudonyms to protect confidentiality.

Researchers' Personal characteristics

During this study, Camilla (MPharmS) was a clinical mental health pharmacist in forensic services in the mental health trust where the study was conducted and the study formed part of her postgraduate MSc Research project. She completed relevant theoretical and experiential training in qualitative research methods, particularly in interpretative phenomenological analysis. CS completed all interviews and focus groups, and was observed on two occasions by Denise Taylor (DT) for appropriateness and effectiveness.

DT (PhD, MSc, MPharmS, FFRPS, MCMHP) was a senior lecturer and research supervisor for CS; providing training in qualitative research methods, analyses, informed consent and capacity assessment.

Relationship with participants

Due to her role as clinical pharmacist, CS had developed professional relationships with some (but not all) of the primary and secondary care HCPs prior to the study; she did not know any of the CSUs. All participants were aware this study was part of a further degree. IPA enables researcher expertise to be acknowledged through reflexivity, whilst simultaneously preserving the voice of the participant.

DAT knew none of the study participants but had previously worked in and researched delivery of clozapine services. Reflexivity throughout the study enabled personal and professional bias to be recognised and addressed.

Data analysis

For peer review only - http://bmjopen.bmj.com/site/about/guidelines.xhtml

As this research explored individuals' experiences and perceptions, interpretative phenomenological analysis (IPA) was used. The intention of IPA is to explore the participants' experience as perceived by them and reflect this in the interpretation by the researcher.[12] In line with accepted analytical process for IPA each transcript was manually coded line-by-line into themes by CS. Robustness of the analytical process was assessed by the coding of a transcript by both CS and DAT. There was a high level of agreement regarding coding and CS continued with the analytical process. At the end of the coding process CS and DAT met to discuss and clarify emerging themes. Once super-ordinate or 'master' themes were identified for each stakeholder participant group, the analysis moved to looking at connections and patterns across all participant groups. No software was used to aid the analysis of data or themes.

RESULTS

A total of thirty-eight participants were recruited; including 32 healthcare professionals (HCPs) and 6 CSU's (General Adult CMHT = 1; Forensic = 5), but no carers as no expressions of interest to participate were received. It is not known how many potential participants who were approached by members of the CMHT and decided not to participate in the study, but once recruited to the study there were no dropouts. 14 interviews and 6 homogenous focus groups from General Adult and Forensic CMHT's were completed. Demographics of participants can be found in Table 3.

Table 3: Demographics of Participants

Participants* per focus group or interview	Profession	Sector	Years with shared-care clozapine
Mark<br>Emily<br>Laura<br>Angela	Hospital Pharmacist<br>MMT[¥]<br>MMT[¥]<br>Receptionist	Hospital Pharmacy, Secondary care	1.5<br>2<br>5<br>5
Luke<br>Bethany<br>John	Community Psychiatric Nurses	Forensic Community Mental Health Team, secondary care	2<br>2<br>2
Kate<br>Anne<br>Denise	Community Psychiatric Nurses	General Adult Community Mental Health Team, secondary care	4<br>4<br>4
Jennifer<br>Trace<br>Victoria	Community Psychiatric Nurse Social Worker Community Psychiatric Nurses	General Adult Community Mental Health Team, secondary care	2<br>5<br>1.5
Lucy<br>Jane<br>Nick<br>Claire	All Social Workers	Forensic Community Mental Health Team, secondary care	2<br>2<br>2<br>2
Tim<br>Simon<br>Adam<br>Frank<br>Rob	All Clozapine Services Users (CSUs	Attached to the Forensic Community Mental Health Team (CMHT)	1-2<br>1-2<br>1-2<br>1-2<br>1-2

For peer review only - http://bmjopen.bmj.com/site/about/guidelines.xhtml

Richard	CSU	Attached to General Adult CMHT	unknown
Dr Harrison	Responsible Clinician	Forensic	2
Dr Brown	Responsible Clinician	Forensic	2
Dr Taylor	Responsible Clinician	Forensic	2
Dr Smith	Responsible Clinician	General Adult	4
Charles	Community pharmacist	Primary care	unknown
Catherine	Community pharmacist	Primary care	2-3
Margaret	Community pharmacist	Primary care	4
Tom	Community pharmacist	Primary care	9 months
Sophie	Community pharmacist	Primary care	unknown
Marie	Community pharmacist	Primary care	4-5
Dr Hudson	GP	Primary care	2
Dr White	GP	Primary care	10
Dr Green	GP	Primary care	2

* Pseudonym names used for confidentiality [¥] Medicines Management Technician

Duration of interviews and focus groups ranged from 30 to 90 minutes and none were repeated. Data saturation was achieved as no new themes emerged during the analysis of final transcripts and field notes were taken for each interview or focus group to support reflexivity. Due to limitations in time and funding, transcripts and findings were not returned to participants for comment. The findings below describe participant perceptions of their experiences of a shared-care clozapine service.

Four superordinate themes emerged from the data; The Sharing of Care, Multi-professional Relationships, The Provision of Care and the Clozapine Process. The inter-connectedness of these themes and minor themes is illustrated in Figure 1.

Excerpts from participant transcripts (shown in indented italic text) have been chosen to exemplify experiential perceptions and changed as little as possible to retain data authenticity. If additions were made for clarification these are bracketed within the quote and non-italicised. When the term Forensic HCP or General Adult HCP is used, Forensic and General Adult CMHT HCP is being referred to respectively.

1. Clozapine Process

For peer review only - http://bmjopen.bmj.com/site/about/guidelines.xhtml

Clozapine is a unique antipsychotic with regard to its prescribing, monitoring and supply; therefore knowledge of all the components of prescribing clozapine and its possible consequences, are important factors in a clozapine supply service.

1.1 Knowledge

The process involved in prescribing and supplying clozapine is different to any other antipsychotic, therefore it takes time and experience to understand and become familiar with its nuances. Greater participant knowledge of the process meant that HCPs understood why things might go wrong, enabling them to have greater confidence in resolving these issues and their role within that.

> *"I looked up some information and used the BNF and it was I guess somewhat hesitant, clearly it is medication that has got some significant potential side effects and risks with it. But umm I think that I felt like I have got a good working relationship with the mental health team and working with the patient that I have on clozapine, and so was able to talk with the* (hospital) *pharmacist and address any issues." Dr Green, GP*

> *"I suppose it's enlarged my experience so I'm more confident with ……once you do more than one customer your confidence increases and you find out the….and you can deal with customers easier can't you? So professionally that way and also you're you feel confident to deal with those patients and other professionals as well" Marie, Community Pharmacist*

1.2 In Practice

Initially HCPs perceived the CSU required certain skills such as organising and planning for shared care clozapine to be successful, so they used surrogate markers of stability and capability to identify suitability of CSUs. With experience of working with shared care, Forensic HCPs realised that CSUs didn't need to be already stable or capable in order for shared care to be successful. They found CSUs responded well to the challenge of being responsible for obtaining their own FBC, repeat prescription and supply of clozapine.

> "[A (CSU) *as well who was really chaotic initially, and we were really worried about whether he'd manage it all, but it's actually made him get a lot more organised." Bethany, Forensic CPN*

HCPs and CSUs perceived the timeframe between obtaining a valid FBC and supplying clozapine before the last supply ran out as being very tight. This became a source of anxiety for both HCPs and CSUs, as they were extremely aware of the potential negative consequences of readmission to hospital for re-titration with a delayed supply of clozapine. Circumstances which could delay the supply of clozapine were perceived to occur relatively frequently by community pharmacists.

> *"….I guess it's just because there's such a tight schedule of the drug, it gives you no leeway for these kind of exceptional circumstances which do happen reasonably regularly." Charles, Community Pharmacist*

Limitations to GP appointment systems were identified, as for some it was not possible to book an appointment 4 weeks in advance for a FBC. This meant CSUs needed to adapt their routine to ensure they remembered to book their appointment at a later date and when sufficient appointments were still available.

For peer review only - http://bmjopen.bmj.com/site/about/guidelines.xhtml

"They give it up to like…I think it's 2 weeks maximum but they won't do a full 28 days, but like (I) write down on a calendar today every month when my calendar starts, need to book the blood test and normally for the week beforehand to book it. " Adam, CSU

2. The Sharing of Care

The sharing of care reverberated far wider than just the prescribing and monitoring of clozapine and was associated with its own obstacles as it required individuals to work in a different way.

2.1 Reverberations of shared care

Some HCPs thought that the drive for shared care clozapine was one of cost saving, however with experience, Forensic HCPs identified greater CSU independence as their driver for engagement. For Forensic HCPs shared care not only enabled CSUs to continue to develop skills required for independence, but also a new opportunity for them to demonstrate trust in the CSU. This trust led to a change in the relationship dynamic and activated the CSU to take responsibility for their care and engage in the process.

"Initially it was savings because….quite some time once I saw how the first person was working and did….once I saw the difference it made with him, how then we felt about him taking medication and how he was responding to that, I think then I moved away from seeing it as a cost saving exercise and seeing it as something that could actually useful in maintaining independence for the client." Nick, Forensic SW

This was supported by CSUs views that shared-care was a supportive opportunity to develop their independence and take back ownership of their health, which they felt in turn boosted their self-esteem. CSUs also perceived that engaging in shared care was normalising and reduced stigma associated with mental illness and clozapine treatment.

"The emphasis is all on you sort of thin in the community, whereas before it was thought that people with mental illness couldn't live in the community properly without having problems and things like that, whereas people are seeing especially the people on Clozapine, that they can live a good normal active life in the community…" Adam, CSU

The physical health of CSUs was valued as equally important to their mental health and shared care provided GP involvement in clozapine prescribing with awareness of the need to manage negative effects of clozapine on physical health. Both Forensic HCPs and CSUs perceived the GP as the expert in physical health and appreciated and benefitted from, this more holistic approach to care. In contrast, GPs not involved in shared care may not even be aware their patient receives clozapine from a mental health trust and any possible associated negative iatrogenic effects. At the time of this study, shared care records between primary and secondary care were not widely used.

"You can't separate off the, you know in the case of these patients, the mental health issues from the physical health and their overall wellbeing, emotional spiritual wellbeing, it is all their whole entity part of the whole and interplay with one another." Dr Green, GP

2.2 Obstacles to working in a different way

For peer review only - http://bmjopen.bmj.com/site/about/guidelines.xhtml

Shared care clozapine required secondary care HCPs to trust primary care HCPs they may not have previously met, and for primary care HCPs to take on the responsibility of an unfamiliar medication with very restrictive prescribing and supply requirements. As mentioned in 1.2, there was initial apprehension on possible negative consequences of a missed clozapine dose. Apprehensions from primary care HCPs eased over time due to growing relationships with secondary care HCPs providing a sense of support and a true sharing of care.

> *"It's not a drug I'm totally at ease with, so there are apprehensions when any specialist says 'please do this, these are the protocols' and unless you really feel you've got a backup, you feel somewhat exposed. That hasn't particularly been an issue because the support has been there." Dr White, GP*

Often General Adult HCPs described negative assumptions on the abilities and/or expectations of CSUs in relation to independence and involvement in managing their own medication. This affected their ability to trust CSUs in taking on this responsibility or even seeing a need for shared care.

> *"I suppose* (it) *depends how well they are doesn't it, how insightful they are in the first place, how sassy they are with how systems work and stuff really. At the end of the day, they just want their meds to turn up when they need more. (The CPN) comes round at 4 with the meds, that's what they want isn't it?" Jane, General Adult CPN*

3. The Provision of Care

Although clozapine is the active treatment, the care provision included the role each member of the multi-professional team played in supporting the recovery and management of the individual.

3.1 Clozapine as a treatment

Many healthcare professionals and all CSUs thought that clozapine often provided the best opportunity for recovery and hospital discharge in individuals with schizophrenia; particularly if they had already tried other antipsychotics without success. These highly valued benefits of clozapine provided the motivation for CSUs to continue taking it despite its negatives effects.

> *"The other thing with Clozapine which was explained to me is side effects and pluses and minuses and sort of thing, but it's like to me it's takin' something that keeps me well. I class it the same as eating food and drinking water. I need that to keep me level and keep me… I feel totally normal now whereas I couldn't before and I was on all of the old medications* [….] *whole range of different and they never really worked. Clozapine was the first one that ever worked. The only downside to it was the side effects; the dribble, makes you sleep, makes you weight gain, things like that but when you weigh up the good and the bad Clozapine is up there it does the job." Adam, CSU*

3.2 Playing my part

Forensic HCPs appreciated the therapeutic value of supporting CSUs to be as independent as possible through normalisation and socialisation. They perceived that a large part of their role was to work with and support CSUs in this way, rather than focussing solely on the therapeutic use of medication.

> *"A large part of our job isn't just about giving medication to people.* (What) *I quite like about my job is about socialisation and normalisation and trying to get people to live in a normal way." John, Forensic CPN*

In contrast, General Adult HCPs perceived a sense of responsibility for co-ordinating the process of clozapine supply and undertook as much of the process as possible on behalf of the CSU. They only saw shared care clozapine as an alternative method of obtaining a supply of clozapine.

> *"Difficult I would say, very difficult, lots of chasing finding about results, seeing if the pharmacy understands you and knows what you're trying to ask for, and then trying to establish whether they've actually got the medication or not, when they're going to get it then you've got to faff around, pick it up and get it to the patient it's a nightmare." Kate, General Adult CPN*

GPs perceived their main role in shared-care to be prescribing clozapine, checking FBC results, and CSU wellbeing and functioning. However they also spent time liaising with primary and secondary care team members with respect to any CSU or clozapine-related problems.

> *"…so there's the practicalities of just the physical monitoring of whether the bloods are ok umm there is a responsibility just to see the patient is ongoing wellbeing and mental health is stable and they're functioning. The responsibility to support the people taking the blood and if they're concerned, they're obviously not qualified, don't have the expertise to make any sort of mental health type assessment, so being available for them to be able to come and say this isn't right worried about this and then obviously responsibility for liaising with the mental health team if there are concerns or worries." Dr White, GP*

In order to supply clozapine on prescription, community pharmacists require all previous steps of the process to be completed. In practice, community pharmacists often found they were heavily involved in identifying and co-ordinating missing elements of the process which prevented them completing their dispensing role.

> *"So we do all the chasing about to find out what stage we're at with all these things because if you phone Denzapine they'll say 'oh we haven't had a result in yet' and then you have to phone the surgery say, 'has this person had a blood test' or get in touch with CPN so we're doing a lot of chasing around." Charles, Community Pharmacist*

CSUs perceived their role in shared-care was to obtain a FBC from the GP surgery, order their prescription, and collect and take, their clozapine. Many CSUs used services provided by community pharmacy such as repeat prescriptions and delivery to support them in undertaking their role in shared care.

> *"Yeah it all good for me now on, so all I've got to do is every 28 days have my bloods done and then they* (community pharmacy) *do the rest and that, so I don't have to sign no paper or run around no more I've just got to go to the GP surgery and see the nurse and have blood samples." Simon, CSU*

4. Multi-professional Relationships

For peer review only

Being recognised as a member of a particular team supported the development of multi-professional relationships and communication. These relationships were integral to building trust and understanding of each other's role and the provision of peer support.

4.1 Recognition as a team member

Knowing the members of the team involved in the provision of shared care clozapine positively influenced communication and supported building relationships with both CSUs and HCPs being more comfortable to make contact with someone if they knew them. This increasing familiarity led to increased involvement and contact which developed relationships still further.

> *"I think if there was a problem with one of my guys in the* (community) *pharmacy where I've been in I'll actually go in and talk to him* (community pharmacist) *now whereas before I'd come through* (the hospital pharmacy) *so I talk to him now.*" *John, Forensic CPN*

Through building a relationship individuals were increasingly able to understand the expertise and role each team member held, which supported identifying the most appropriate person to contact for support.

> *"I mostly do things to do with my mental health with my consultant and things to do with my physical health with my GP." Richard, CSU*

4.2 Supporting each other

Relationships were a key element in feeling supported and building trust. Support ranged from provision of information or advice to provision of additional resource when needed. The sense of feeling supported was characterised through a reliable relationship which was responsive to need.

> *"Knowing that there is somewhere that you can call on is important and certainly the particular community mental health nurse who is involved in the care she is excellent. Apart from these meetings she is available for discussion and she has come along and organised blood tests and like if there seems to be a more urgent situation where some compliance has gone wrong."*
> *Dr White, GP*

Sharing the responsibility of clozapine provision through shared-care was seen as a source of support for primary care by GPs and RCs, as it reinforced a sense of an equally shared responsibility and supported the ability to work in a multi-disciplinary way.

> *"Well it enables us to umm to manage this difficult client group for patients that need to take it, so we are happy to accept our share of the responsibility for that and…. so it is a positive thing and it helps us with our sort of multi-disciplinary work."* *Dr Hudson, GP*

DISCUSSION AND CONCLUSIONS

Principle findings

All participants perceived clozapine provision as different to other antipsychotics because of the process of supply, its beneficial and negative effects, greater reliance on members of the multi-

For peer review only - http://bmjopen.bmj.com/site/about/guidelines.xhtml

professional team and system processes. Greater knowledge and understanding of clozapine provision was generally obtained through experience and led to individuals having more confidence in their own role and resolving issues that arose. Developing multi-professional relationships was key in building trust, understanding roles, improving communication and feeling supported. Shared care reinforced a sense of equal and shared responsibility between primary and secondary care; this supported further development of multi-professional relationships and was seen as a source of support. Addition of a GP to the team, provided more holistic care for the CSU enabling physical health interventions and improved GP awareness that their patients were taking clozapine. This awareness decreased potential iatrogenic health risks associated with clozapine.[13]

Surrogate markers of stability and capability were originally used to identify CSUs who were potentially suitable for clozapine shared care. However with experience Forensic HCPs identified shared care as a new opportunity to demonstrate trust in the CSU and support them to develop skills of self-dependence and independence through normalisation and socialisation. This reduced Forensic HCPs reliance on surrogate markers. Engaging in shared care clozapine led to a change in the relationship dynamic between the Forensic HCPs and CSUs, which in turn enabled the CSU to take responsibility and ownership for their care. In contrast, General Adult HCPs held paternalistic assumptions of CSU abilities and/or expectations in relation to their independence and involvement in managing their own medication. This affected General Adult HCPs ability to trust CSUs in possibly taking on this responsibility or perceiving a need for shared care clozapine.

Implications for Practice

Relationships in healthcare provision

In 2010, the Health Foundation launched 'Closing the Gap through Changing Relationships', a programme which focused on recognising the need to change the way healthcare systems work by challenging beliefs and behaviours of healthcare workers to improve quality of care. Evidence demonstrates that best health outcomes and experiences are achieved when people have an active part in their own care and receive responsive support according to their needs. Being able to play an active role is a consequence of the dynamic created by the manner in which support is provided.[14] The programme also explored relationships between service users and providers in healthcare settings using a conceptual framework proposed by Wish et al,[14,15] which identified four primary relationship dimensions to describe the character of different relationships. These are power, valence, intensity and formality.[15]

We mapped the HCP-CSU relationships in Forensics and General Adult to the above relationship dimensions to explore how shared care clozapine influenced these HCP/CSU relationships. See Table 4 for further detail.

For peer review only

Table 4: Mapping Relationships to the Wish Model

The Wish Model Relationship Continuum Components		The Relationship Dynamic	
		**Forensic CMHT HCPs**	**General Adult CMHT HCPs**
Symmetric --- Asymmetric	**Power**<br>dependence<br>centredness<br>agency	Greater sense of partnership for both CSUs and HCP.<br><br>Themes centred around the CSU rather than the HCP.<br><br>CSUs have gained greater agency and reduced dependence on HCPs.<br><br>**Result: perception of a symmetrical HCP/CSU relationship.**	HCPs complete activities either for or on-behalf of CSU.<br><br>Themes centred on HCPs rather than CSU.<br><br>CSU appears to be highly dependent on the HCP.<br><br>**Result: perception of an asymmetrical HCP/CSU relationship.**
Cooperative --- Competitive	**Valence**<br>agreement<br>communication<br>conflict	HCP & CSU agendas aligned to increase independence and normalisation.<br><br>Engagement in shared care provided an opportunity to achieve their shared agendas.<br><br>HCPs trust in CSUs ability to perform own role in shared care.<br><br>**Result: perception of a co-operative HCP/CSU relationship.**	No evidence of agenda alignment.<br><br>Formal medical knowledge competing with lay knowledge.<br><br>Some HCPs described lacking trust in CSU relationships.<br><br>**Result: perception of a competitive component to HCP/CSU relationship.**
Intimate --- Distant	**Intensity**<br>transactional/ relational<br>commitment	Monitoring prescription collection transactional element but in context of a relational focus on a CSU long-term goals.<br><br>Forensic HCPs demonstrated commitment through motivation and willingness to adapt their beliefs and behaviours to fulfil the shared agenda of independence. CSUs committed to their own role in shared care.<br><br>**Result: perception of an intimate HCP/CSU relationship.**	Adult HCPs talked about undertaking activities on behalf of the CSU with no reference to the future aims of the CSU.<br><br>Adult HCPs demonstrated little change in beliefs and behaviours in a shared care role of the CSU's; suggesting a distant relationship with little commitment for the potentially longer term goals of the CSU.<br><br>**Result: perception of a transactional and distant HCP/CSU relationship.**
Social --- Professional	**Formality**<br>affect<br>exclusivity	Engaging in shared care enabled a shift in formality dimension to social as the relationship extends into primary care.<br><br>Social affect aided by the commitment to HCP-CSU relationship.<br><br>**Result: perception of a more social professional HCP/CSU relationship.**	Considerable professional formality.<br><br>Professional affect demonstrated by the power differential between the HCP and CSU.<br><br>**Result: perception of a formal professional HCP/CSU relationship.**

For peer review only

General Adult HCPs appeared to hold negative assumptions of the abilities and/or expectations of CSUs managing their own medication. These assumptions reinforced their inherent paternalistic role demonstrated by taking responsibility for the CSU obtaining clozapine, which created dependence. Shared care clozapine appears to have had no influence in supporting changes in the General Adult HCP-CSU relationship dimensions and so it continues to be characterised by asymmetric, competitive, distant and professional qualities. This may provide an explanation as to why General Adult HCPs did not appear to see a need for shared care clozapine or invite CSUs to participate in this study.

In contrast, the effect of shared care clozapine on Forensic HCP-CSU relationship, influenced change in all four Wish dimensions. Engaging in shared care clozapine enabled a shift in the power differential between HCP and CSU to a more symmetric and co-operative approach, possibly through the ability to trust in each other and share a common agenda of CSU independence. The willingness to engage in new ways of working, enabled them to tailor their support for CSUs to continue to develop skills for independence, demonstrating commitment to the long term agenda of independence and relationship integrity. Shared care clozapine appeared to cultivate changes in the Forensic HCP-CSU relationship dimensions which enabled CSUs to expand their ability to live independent and fulfilling lives; key components of person-centred care.[16] In contrast the General Adult HCP-CSU relationship is reflective of a biomedical l model and they described a more negative experience of shared care compared to the more positive experience of Forensic HCPs.[17]

Person-centred care

Providing care which is person-centred is dependent on ensuring care affords people dignity, respect and compassion; care, support or treatment which is coordinated, personalised and enables patients to live an independent and fulfilling life.[16]

Key principles of person-centred care with particular relevance to successful shared care clozapine outcomes include knowing the patient as a person, recognising their individuality and expertise in their own health and care and taking a holistic approach to assessment and provision of care which is centred around them. Importantly staff need to be supportive, share the power and responsibility of care and be well trained in communication.[18]

Engagement in shared care clozapine with a relationship demonstrated by the Forensic HCP/CSU partnership, provides an example of a shift in care from paternalistic to person-centred. We suggest that shared care clozapine in this context, offers an opportunity to demonstrate dignity, respect and compassion to CSUs through trust and handing over greater responsibility to the CSU. The provision of coordinated care and support through the development of relationships between multi-professional team members in primary and secondary care enabled personalised care and support for CSUs, which was subsequently reflected in CSUs developing their ability to manage their own role in shared care. This person-centred approach ultimately enabled CSUs to live an independent and fulfilling life by self-managing their own clozapine.

Dartmouth Atlas defines effective care as *"services that are of proven value and have no significant tradeoffs"*[19] Based on CSUs and Forensic HCPs experiences, shared care clozapine is effective for these participants. Currently there is no definition of acceptability in healthcare, although Sekhon et al (2017), propose a theoretical yet to be validated framwork.[20] We suggest that acceptability of shared care clozapine is demonstrated by the active engagement of CSUs and Forensic HCPs in the process.

For peer review only - http://bmjopen.bmj.com/site/about/guidelines.xhtml

Future research

Future research using implementation theory is needed to understand in greater detail, the barriers and enablers to wider implementation of shared care clozapine to support integration of people with serious mental illness into the community.[21,22]

Strengths and limitations of this study

This is the first qualitative study to explore individual perceptions on experiences of shared-care clozapine service by those that use and provide it. Identification of potential participants relied on two information sources being up-to-date; the Denzapine® Monitoring Service website and electronic medical notes. CSU participants were not equally represented from Adult and Forensic CMHTs, which may mean experiences and perceptions of General Adult CSUs are not sufficiently represented. Recruitment of CSUs was dependent on invitation for participation from their own clinical team. Greater recruitment of Forensic CSUs could relate to their enabling approach to care or a reflection of the team's experience of shared-care in comparison to General Adult CMHT. No carers of CSUs participated, possibly reflecting the isolation that some people with serious mental illness live with.

Interpretative Phenomenological Analysis methodology guided the development of topic guides, completion of interviews and homogenous focus groups used to collect the qualitative data. IPA also allowed the experience of the researchers to be noted in the reflexivity associated with data analysis. Quality assurance processes were completed to provide validity to this research, for example piloting topic guides, supervised focus groups and joint co-coding and re-coding of data. Data validity is reflected in the ability to substantiate the analysis with participant quotes and provide a dialogue of discussion between the results and existing literature.

Conclusions

Mental health has seen an increase in demand on limited health service resources with cuts in funding. This has led to a number of trusts embarking on transformative programmes in order to reduce costs, shift demand from acute services, and deliver care focused on recovery and self-management.[23] Person-centred care is recognised nationally as one way in which this can be achieved through a substantial effect on quality of care.[16,18] Our results suggest shared care clozapine could be one way of expanding person-centred care in mental health which the literature demonstrates demonstrable improvements in quality of care, integration into the community and reductions in stigma and healthcare resources.

Figure 1: Illustration of superordinate themes and their connections

Acknowledgements

This study was funded by a small grant from the College of Mental Health Pharmacy. We thank all study participants for their input into this study and for the team members who helped in identification of potential participants.

For peer review only

REFERENCES

1 DH. *Equity and excellence: Liberating the NHS*. July 2010.

2 HMG/DH. *No Health Without Mental Health: A Cross-Government Mental Health Outcomes Strategy for People of All Ages*. February 2011.

3 NHS England. *Five Year Forward View*. October 2014.

4 Cowen P, Harrison P, Burns T. *Shorter Oxford Textbook of Psychiatry* (Chapter 11, Schizophrenia). Oxford University Press, 2012: 255-296.

5 Kane J, Honigfeld G, Singer J, Meltzer H, The Clozaril Collaborative Study Group. Clozapine for the treatment-resistant schizophrenic: results of a US multicentre trial. *Psychopharmacology*. 1989; 99: S60-S63.

6 Britannia Pharmaceuticals. *Denzapine 100mg tablets - Summary of Product Characteristics* (15th January 2015). http://www.medicines.org.uk/emc/medicine/25965 (accessed 11 Jun 2017).

7 Filia S, Lee S, Sinclair K, Wheelhouse A, de Castella A et al. Demonstrating the effectiveness of less restrictive care pathways for the management of patients treated with clozapine. *Australasian Psychiatry*. 2013; 21(5): 449-455.

8 Filia S, Wheelhouse A, Lee S, Main M, de Castella A, Wilkins S et al. Transitioning patients taking clozapine from the public to private/GP shared-care setting: Barriers and criteria. *Australian and New Zealand Journal of Psychiatry*. 2012; 46(3): 225-231.

9 Kelly B, Perkins D, Fuller JD, Parker S. Shared care in mental illness: A rapid review to inform implementation. *International Journal of Mental Health Systems*. 2011; 5: 31.

10 Smith SM, Allwright S, O'Dowd T. Effectiveness of shared care across the interface between primary and speciality are in chronic disease management. *Cochrane Database of Systematic Reviews*. 2007.

11 Smith JA, Osborn M. Chapter 4 *Interpretative Phenomenological* Analysis in Qualitative Psychology. A Practical Guide to Research Methods. Ed Smith JA. Sage Publications, London 2003.

12 Smith J, Flowers P, Larkin M. *Interpretative phenomenological analysis: Theory, Method and Research*. Sage, 2013.

13 Murphy K, Coombes I, Moudgil V, Patterson S, Wheeler A. Clozapine and concomitant medications: Assessing the completeness and accuracy of medication records for people prescribed clozapine under shared care arrangements. *J Eval Clin Pract.* Published Online First: 4 May 2017. Doi: 10.1111/jep.12743

14 The Health Foundation. *Closing the gap through changing relationships: evaluation*. June 2014. London, The Health Foundation. http://www.health.org.uk/sites/health/files/ClosingTheGapThroughChangingRelationshipsEvaluation.pdf (last accessed20-06-2017)

15 Wish M. Comparisons among multidimensional structures of interpersonal relations. *Multivariate Behav Res*. 1976; 11(3): 297-324.

16 The Health Foundation. *Person-centred care made simple*. 2014. London. The Health Foundation. http://www.health.org.uk/sites/health/files/PersonCentredCareMadeSimple.pdf (Last accessed 20-06-2017)

17 Engel G. The Need for a New Medical Model: A Challenge for Biomedicine. *Science*. 1977; 196: 129-196.

18 Health Innovation Network: South London. What is person-centred care and why is it important?. http://www.hin-southlondon.org/system/ckeditor_assets/attachments/41/what_is_person-centred_care_and_why_is_it_important.pdf (accessed 11 Jun 2017).

19 Anon. Effective Care. A Dartmouth Atlas Project Topic Brief. May 2007. Centre for Evaluative Clinical Services. http://www.dartmouthatlas.org/downloads/reports/effective_care.pdf (accessed 20 06 2017).

20 Sekhon M, Cartright M, Francis J. Acceptability of healthcare interventions: an overview of reviews and development of a theoretical framework. *BMC Health Services Research.* Published Online First: 26 January 2017. doi: 10.1186/s12913-017-2031-8

21 May C, Finch T. Implementing, Embedding and Integrating Practices: An Outline of Normalization Process Theory. *Sociology*. 2009; 43(3): 535-554.

22 Greenhalgh T, Robert G, MacFarlane F et al. Diffusion of innovations in service organisations: Systematic review and recommendations. *The Milbank Quarterly*. 2004; 82(4): 581-629.

For peer review only

For peer review only - http://bmjopen.bmj.com/site/about/guidelines.xhtml

23 The Kings Fund. Briefing: Mental health under pressure. https://www.kingsfund.org.uk/sites/files/kf/field/field_publication_file/mental-health-under-pressure-nov15_0.pdf (accessed 11 Jun 2017).

For peer review only

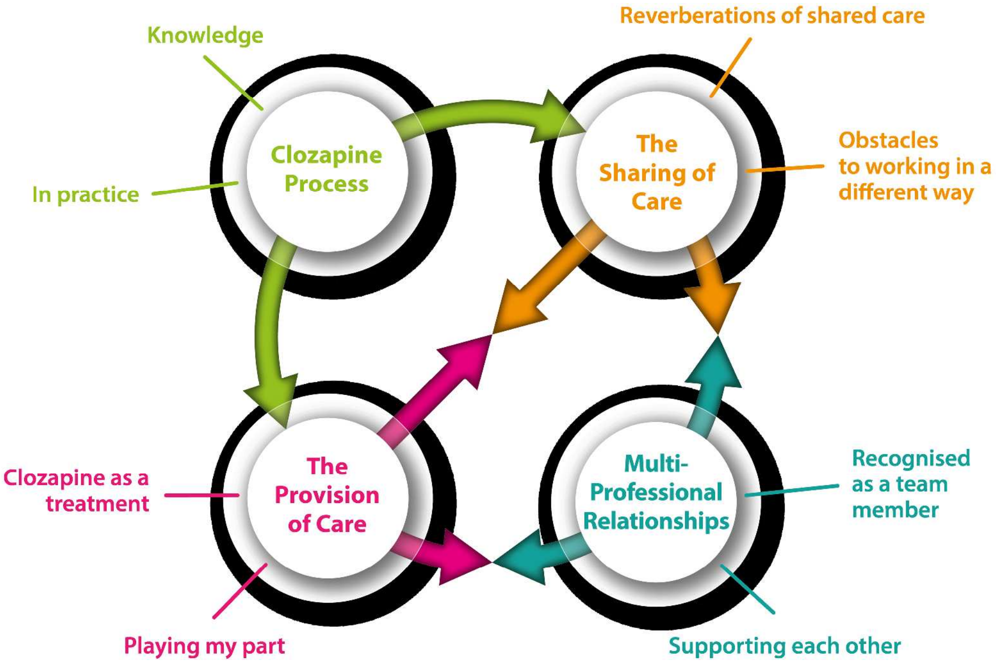
Knowledge
Reverberations of shared care
Clozapine Process
In practice
The Sharing of Care
Obstacles to working in a different way
The Provision of Care
Clozapine as a treatment
Multi-Professional Relationships
Recognised as a team member
Playing my part
Supporting each other

eview only
